# Integration of mRNA and miRNA profiling reveals the heterosis of three hybrid combinations of *Capsicum annuum* varieties

**DOI:** 10.1080/21645698.2020.1852064

**Published:** 2021-01-07

**Authors:** Sha Yang, Zhuqing Zhang, Wenchao Chen, Xuefeng Li, Shudong Zhou, Chengliang Liang, Xin Li, Bozhi Yang, Xuexiao Zou, Feng Liu, Lijun Ou, Yanqing Ma

**Affiliations:** a Institution of Vegetable Research, Hunan Academy of Agricultural Science, Changsha, Hunan, China; b College of Horticulture, Key Laboratory for Vegetable Biology of Hunan Province, Hunan Agricultural University, Changsha, Hunan, China; c Department of Agriculture and Rural Affairs of Hunan Province, ChangshaHunan, China

**Keywords:** *Capsicum annuum*, rna-seq, gene regulation, cross combination, heterosis

## Abstract

*Capsicum annuum* is also known as chili which is one of the most important vegetable crops grown in the world. Breeding new varieties with heterosis could improve the quality of pepper, increase yield, growth potential, disease resistance, adaptability, and seed viability. To investigate the heterosis among three cross combinations of different parents, the mRNA-miRNA integrated analysis was performed. A total number of 22,659,009 to 36,423,818 clean data were generated from mRNA-seq with 81 libraries, and the unique mapped reads were from 35,495,567 (86.81%) to 46,466,622 (88.95%). The plant-hormone signal transduction pathway (40 genes) was detected with a higher DEG number. The *SAUR32L, GID1, PYR1, EIN2. ERF1, PR1, JAR1-like, IAA* from this pathway play a key role in plant development. From the miRNA-seq, the number of clean reads was ranging from 12,132,221 to 25,632,680. A total of 220 miRNAs were predicted in this study, and all of them were identified as novel miRNA. The top three candidate KEGG pathways of miRNA were ribosome signaling pathway (13 miRNAs), spliceosome pathway (13 miRNAs), and plant hormone signal transduction pathways (10 miRNAs). With the mRNA and miRNA integrated analysis, we found some key genes were regulated by some miRNAs. Among them, the scarecrow-like 6 protein can be up or down regulated by mir8, mir120, mir184, mir_214, mir125, and mir130. The function of Della protein was regulated by mir24, mir74, mir94, mir139, and mir190. This study contributes to understanding how heterosis regulates the traits, such as crop production, fruit weight, and fruit length.

## Introduction

*Capsicum annuum*, known as chili, cayenne pepper, sea pepper, and bell pepper, is a therophyte plant in the temperate zone and perennial shrub in the tropical zone. It is one of the most important vegetable crops grown in the world.^1–3^ China’s capsicum production in 2017 achieved 17,821,2383 tons.^4^ *C. annuum* was used as spices in most of the cuisine to enhance the aroma and taste of the food. The *C. annuum* can be consumed as fresh or dried^5^, and dried *C. annuum* can be store for a longer time. *C. annuum* has a variety of bioactive compounds including capsaicin which showed great pharmaceutical and antimicrobial benefit.^6,7^ The capsaicin in *C. annuum* showed therapeutic ability such as anticancer agent, antiobesity, cardiovascular effect, dermatological effect, and neuropathic pain relief.^4^ *C. annuum is* native to Mexico, Peru, and other countries in the tropical region of Central and South America. It was introduced into China in the late Ming dynasty and then widely planted. *C. annuum* has become one of the largest vegetable crops in China.^8^ The pepper-consuming population in China was mainly centralized in Sichuan, Guizhou, Hunan, and Hubei provinces.^5^ At present, although there are many species of pepper in China, the types are relatively concentrated, and many species showed homology and anonymity. Therefore, it is particularly important to breed new pepper varieties with optimal traits. The breeding experience has revealed that breeding new varieties with heterosis is an effective way to solve this problem. It is not only improving the quality of pepper, increase yield, growth potential, disease resistance, adaptability, and seed viability.^8^ Hence, it is significant to improve the yield and quality of pepper by using prediction and parental selection. Heterosis is a ubiquitous phenomenon in the biological world. Heterosis is one of the most widely used and effective breeding methods in most plants.^9^ The *C. annuum* hybrid ensures the high yielding capacity in terms of fruit length and weight, shorten maturation period, and a higher number of fruits produced per plant.^10^ Hybrid F1 represents the phenomenon of superiority over parents, greatly promotes the development of agricultural production.^11^ Since the past decades, the hybridization of common pepper has been a hot research topic. Manzur et al. conducted the wide hybridization between *C. annuum* and *C. baccatum* and provided breeders with useful practical information for the regular utilization of the *C. baccatum* gene pool in *C. annuum* breeding.^12^ Pathy et al. performed three-way Cross and double-cross hybrids in *C. annuum* and accessed the breeding potential of multi-parent crosses.^13^ Although the genome data of *C. annuum* has been published,^14^ little information about the genetic regulation of heterosis is available.

Next-generation sequencing (NGS) including RNA-seq and miRNA-seq technologies enables the researcher to study the comprehensive gene transcription process, gene-regulating network, and molecular mechanism systematically.^15^ RNA-seq technique has been used to discover the differential expression profiles and revealed the signaling transduction pathways involved in the biological process.^16,17^ Transcriptomic analysis has been widely performed in many plant species such as Arabidopsis^18^; Sorghum^19^; Cotton^20^; *Brassica campestris*,^21^ as well as *C. annuum*.^22^ Chen et al. used the RNA-seq method to compare the sterile and fertile plant of *C. annuum* and found 668 genes were differentially expressed.^23^ Likewise, Li et al. performed comparative transcriptome analysis of heat-susceptible and heat-tolerant *C. annuum* and found some genes that involved in stress response were up-regulated under heat stress.^24^ Li et al. used RNA-seq to determine the molecular roles of 24-epibrassinolide (EBR) during a chilling stress response and revealed that the brassinosteroids could induce the tolerance to chilling stress in pepper.^25^ The miRNAs are an endogenous non-coding small RNA (ranging from 21 to 24 nucleotides). The miRNAs were proved to function in the growth and reproduction process in plant.^26^ Meanwhile, miRNAs can regulate developmental phase transition, metabolism, stress response, and hormonal signaling in plants.^27–29^ The plant miRNAs can repress translation through a slicer-independent mechanism.^30^ A previous study has identified 128 conserved miRNAs of *C. annuum* and found that the novel target of miR-365 was involved in RNA-directed DNA methylation in plants.^31^ Liu et al. also identified a total of 59 known and 310 novel miRNAs using RNA-seq and 656 target genes were predicted which were involved in starch sucrose metabolism and amino sugar metabolism.^32^

In this study, 81 samples including three tissues (flower bud, young fruit stage-1, and young fruit stage-2) and 27 miRNA libraries including 6 parental strains and 3 hybrid cross combinations were sequenced. This study aimed to reveal the main and major signaling pathway which involved in the heterosis of the 3 hybrids combination by integrating mRNA and miRNA profiling analysis. Besides, we analyzed and determined the major miRNAs and functional genes from the mRNA-miRNA analysis, to find out the regulation and transcription level of these genes in the three hybrid cross combinations.

## Materials and Methods

### Experimental Design

**Figure 1.**
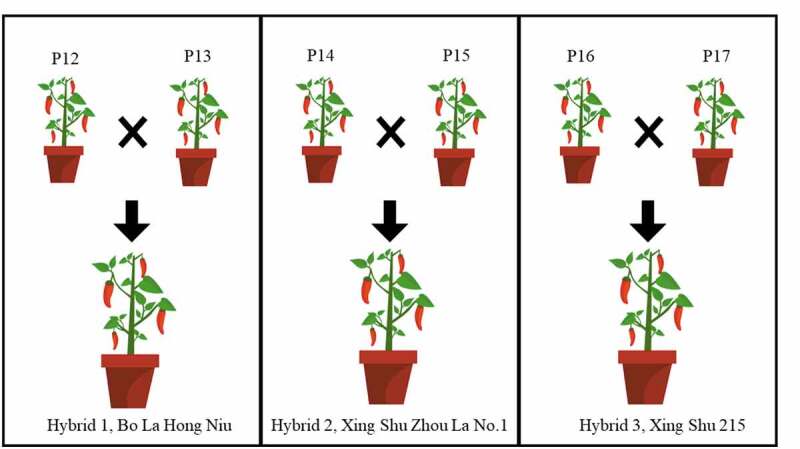
The three groups of pepper cross combination with their parental strains

The parental pepper was planted at Hunan planting base. A total of 6 parental pepper strains was selected to generate three hybrid combinations. The 6 parental strains were named as P12, P13, P14, P15, P16, and P17, respectively. To obtain Bo La Hong Niu (Hybrid 1), P12 (SF-11-1) as a female parent while P13 (SJ05-12-5) as a male parent. The P12 (SF-11-1) strain was obtained from Loudi, showed early maturity, 16.8 cm and 1.4 cm of fruit length and fruit width respectively, the fruit surface is bright and slightly wrinkled, and have strong disease resistant. P13 (SJ05-12-5) strain was obtained from Jiangxi, is an excellent inbred line with mid-early maturation feature, 18.0 cm and 1.6 cm of fruit length and fruit width, and have shiny and slightly wrinkled fruit surface. To obtain Xing Shu Zhou La No.1 (Hybrid 2), P14 (SJ07-116) as a female parent while P15 (H1023) as a male parent. P14 (SJ07-116) strain was obtained from Liuyang, showed early maturity, strong branching ability, good resistance to low temperature, 20.5 cm and 2.7 cm of fruit length and fruit width, and have green and bright fruit surface. The P15 (H1023) strain was obtained from Anhui, have a strong spicy taste, good disease resistance, fast growth rate, 22.5 cm and 2.6 cm of fruit length and fruit width, and the fruit surface is dark green and wrinkled surface. To obtain Xing Shu 215 (Hybrid 3), P16 (2144) as a female parent while P17 (8214) as a male parent. P16 (2144) strain was obtained from Hexi and has better disease resistance, 17.2 cm, and 2.4 cm of fruit length and fruit width, and has green fruit skin. P17 (8214) strain was obtained from Xiangtan, showed mid-to-late maturation feature and 15.8 cm and 2.2 cm of fruit length and fruit width. The combination of a cross to generates hybrid heterosis was P12 × P13, P14 × P15, and P16 × P17 (Fig. 1). The physical and morphological parameters of parental strains and F1 hybrids, including first flower node, main stem height, number of fruits per plant, single fruit weight, plant height, plant size, fruit length, fruit width, and pulp thickness was measured.

### Total RNA Isolation and Library Construction

The total RNA of flower bud, young fruit (stage 1 and stage 2) of three hybrid groups were collected and isolated immediately using RNAiso plus (TaKaRa, Dalian, China). The RNA concentration was checked using Nanodrop 2000 spectrophotometer (Thermofisher, USA). RNA integrity was assessed using the RNA Nano 6000 Assay Kit of the Agilent Bioanalyzer 2100 system (Agilent Technologies, CA, USA). For RNA-seq library constructions, 1 μg RNA per sample was used as input material for the RNA sample preparations. Sequencing libraries were generated using NEB NExt UltraTM RNA Library Prep Kit for Illumina (NEB, USA) by following the manufacturer’s protocols. For miRNA-seq library constructions, the RNA samples were ligated with the 3ʹ SR and 5ʹ SR adaptor. The reverse transcription synthetic the first chain by using NEB Next Ultra-small RNA Sample Library Prep Kit for Illumina (NEB, USA). Polyacrylamide gel electrophoresis (PAGE) gel was used to electrophoresis fragment screening purposes, rubber cutting recycling as the pieces get small RNA libraries. The obtained PCR products were purified using the AMPure XP system and library quality was assessed.

### Sequencing and Functional Annotation

The 81 mRNA libraries were sequenced on an illumine Hiseq 2500 platform with a 150 bp paired-end. The clean reads were obtained by removing reads containing adapter, reads containing poly-N and low-quality reads from raw data. Hisat2^33^ and Stringtie^34^ were used to map with reference genome.^14^ Genes were annotated using BLAST against the public databases, including Nr (NCBI non-redundant protein sequences), Nt (NCBI non-redundant nucleotide sequences), Pfam (Protein family database), KOG (The database of Cluster of Protein homology,), COG (Cluster of Orthologous Groups of proteins), Swiss-Prot (A manually annotated and reviewed protein sequence database), KEGG (The database of Kyoto Encyclopedia of Genes and Genomes), GO (Gene Ontology). Quantification of gene expression levels was estimated by fragments per kilobase of transcript per million fragments mapped (FPKM).

Differential expression analysis of two conditions/groups was performed using DEseq.^35^ DEseq provides statistical routines for determining differential expression in digital gene expression data using a model based on the negative binomial distribution. The *P* values were adjusted using Benjamini and Hochberg’s approach to control the false discovery rate. Genes with an adjusted *P*-value < 0.01 and fold change > 2 were defined as differentially expressed genes. Gene Ontology (GO) enrichment analysis of the differentially expressed genes (DEGs) was implemented by the GOseq R packages based on Wallenius non-central hypergeometric distribution,^36^ which can adjust for gene length bias in DEGs. KOBAS software^37^ was used to test the statistical enrichment of DEGs in KEGG pathways.

### The microRNA Sequencing and Bioinformatic Analysis

The miRNA library preparations were sequenced on an illumine Hiseq platform with 50 bp single ends. The clean data was obtained by removing the reads containing adapter, read containing poly-N and low-quality reads from raw data. Then, the clean reads were trimmed by removing the sequences smaller than 18 nt or longer than 30 nt. The obtained clean reads were mapped using the Bowtie2 program to the databases, including the Silva database (http://www.arb-silva.de/), GtRNAdb database (http://lowelab.ucsc.edu/GtRNAdb/), Rfam database (http://rfam.xfam.org/) and Repbase database sequence alignment (http://www.girinst.org/repbase/). The ribosomal RNA (rRNA), transfer RNA (tRNA), small nuclear RNA (snRNA), small nucleolar RNA (snoRNA), and other ncRNA and repeats were annotated. The remaining reads were used to identified known miRNA with miRbase and novel miRNA. Randfold (v2.0) was used for secondary structure predictions of novel miRNAs. Based on known and novel predicted miRNAs and gene sequence information of corresponding species, TargetFinder software^38^ was used to predict target genes in pepper.

Differentially expressed miRNAs were detected using the DEseq2 package (v1.10.1).^39^ DEseq2 provides statistical routines for determining differential expression in digital gene expression data using a model based on the negative binomial distribution. The miRNA with |log2(FC)|≥1; FDR≤0.05 was assigned as differentially expressed miRNA. The *P*-value was adjusted using the q value. The |log2(FC)|≥1; FDR≤0.05 was set as the threshold for significantly differentially expressed. The Gene Ontology (GO) enrichment analysis of the DEGs was implemented by the GOseq R packages based on Wallenius’ non-central hypergeometric distribution. KOBAS^37^ software was used to test the statistical enrichment of DEGs in KEGG pathways.

### The mRNA-miRNA Integrated Analysis

According to the miRNA sequencing and transcriptomic sequencing, the differentially expressed miRNAs and mRNAs in two groups or two samples were searched. The relationships between differentially expressed miRNAs and differentially expressed genes were searched according to the regulation effect of miRNAs on the RNA. Due to the negative regulatory effect of miRNAs on the RNA, the miRNAs and RNA pairs with negative regulatory network relationship were mainly analyzed

### Quantitative RT-qPCR Assays for mRNA and miRNA Transcriptions

The RT-qPCR primers of selected genes in mRNA category, *SAUR32L* (SAUR family protein), *GID1* (gibberellin receptor 1), *PYR1* (abscisic acid receptor PYR/PYL family 1), *EIN2* (ethylene-insensitive protein 2), *ERF1* (ethylene-responsive transcription factor 1), *PR1* (pathogenesis-related protein 1), *JAR1-like* (jasmine acid-amino synthetase 1-like), and *IAA* (auxin-responsive protein IAA) were designed. The *β-actin* gene was used as internal reference genes. For the miRNA verification, the primers of miR-11, miR-59, miR-86, and miR-128 were designed and *Cp-actin* was used as an internal reference gene. All the primers for mRNA and miRNA quantification were listed in Table 1. Total RNA of samples was isolated with RNAiso plus (TaKaRa, Dalian, China) and reverse transcript to single-strand cDNA by using PrimeScript RT Reagent Kit with genomic DNA (gDNA) eraser (TaKaRa, Dalian, China) according to the manufacturer’s instructions. The expression patterns of selected genes were performed on the Quantstudio 6 Flex (Applied Biosystems, Thermo Fisher, USA). The RT-qPCR was performed in a 96-well plate, with each well containing 20 μl of reaction mix containing 10 μl SYBR PreMix Ex Taqm II (TaKaRa, Dalian, China, 0.4 μl each of the forward and reverse primers. (10 μM), 2 μl complementary cDNA template of each sample, and 7.2 μl sterilized double-distilled water (ddH_2_O). The RT-qPCR conditions were pre-denaturation at 95°C for 5 mins, followed by the 40 cycles of amplification at 95°C for 15s, 60°C for 45s, and 72°C for 15s. All of the genes of each sample were analyzed performed triplicate. The expression of each gene of each sample was calculated using 2^−ΔΔCt^ methods.

**Table 1.** List of the primers used for mRNA and miRNA gene transcription analysis by qPCR

Names	Sequence (5ʹ to 3ʹ)
*SAUR32L*-F	GGTTACTTTGCTGTATGCTCAGT
*SAUR32L*-R	AGTAATTGCAAGAATGAAGGGTCA
*GID1*-F	GGTGGACAAGAGAGAACAGAAT
*GID1*-R	CAGGAAGATAGGCTCTCCAATAC
*PYR1*-F	GAAGGGAATACGGAGGAAGATAC
*PYR1*-R	CCGGCCATAGTTTCAGTTACA
*EIN2*-F	GGAAGGATCCGAGTGGTTATTT
*EIN2*-R	CTCCCTAGTTTCAGCATCATAGAG
*ERF1*-F	CGGCGGAAATAAGGGATTCA
*ERF1*-R	CGTACGCAGCTTGGTCATAA
*PR1*-F	AGAGCTACTCAGCCACATCT
*PR1*-R	CACATCTTTCCCTCTCTGGATTAC
*JAR1-like*-F	TCGTTCGTTGATGCAGGATAC
*JAR1-like*-R	GGCTAACAGCACCTCCTAATC
*IAA*-F	GCTTCGAACTGTGAGGGCAG
*IAA*-R	AGGCAGAGGTTATTGTGTTCG
*β-actin*-F	TGCAGGAATCCACGAGACTAC
*β-actin*-R	TACCACCACTGAGCACAATGTT
miR-11-F	ACACTCCAGCTGGGTTTGATGCTCTTTGT
miR-11-R	CTCAACTGGTGTCGTGGAGTCGGCAATTCAGTTGAGTGTCAAAC
miR-59-F	ACACTCCAGCTGGGTTCTTGGCTAGAGTTG
miR-59-R	CTCAACTGGTGTCGTGGAGTCGGCAATTCAGTTGAGGCAACACA
miR-86-F	ACACTCCAGCTGGGTGAAGCTGCCAGCAT
miR-86-R	CTCAACTGGTGTCGTGGAGTCGGCAATTCAGTTGAGTAGATCAT
miR-128-F	ACACTCCAGCTGGGTTAGCAACAACAATA
miR-128-R	CTCAACTGGTGTCGTGGAGTCGGCAATTCAGTTGAGCATATATA
*Cp-actin*-F	CCACCTCTTCACTCTCTGCTCT
*Cp-actin*-R	ACTAGGAAAAACAGCCCTTGGT

### Statistical Analysis

All collected quantitative data presented as the means of three individual experiments with standard errors (SE). Collected data were analyzed by using IBM SPSS 19.0 software (IBM Analytics, Richmond, VA, USA). The significant differences among samples were analyzed by one-way analysis of variance (ANOVA) using the least significant difference (LSD) multiple range test. Two significant thresholds were applied, *P* < .05 was a statistically significant difference, and *P* < .01 was a highly significant difference.

## Results

### Morphological Comparison of Three Cross Combination

The morphological parameters of 6 parental strains and 3 F1 hybrids plants were measured and recorded. A total of 9 external parameters consisting of first flower node, main stem height, number of fruits per plant, single fruit weight, plant height, plant size, fruit length, fruit width, and pulp thickness were measured (Table 2). The hybrid progenies plants showed a higher number of fruits yield per plant compare to parental plants. In addition, the hybrid progenies have heavier fruit weight and longer fruit length. The hybrid progenies did not show any significant difference in the fruit width and pulp thickness.

**Table 2.** The physical and morphological measurement of parental and hybrid progenies

Parameters	P12 (cm)	P13 (cm)	Hybrid 1 (cm)	P14 (cm)	P15 (cm)	Hybrid 2 (cm)	P16 (cm)	P17 (cm)	Hybrid 3 (cm)
First flower node	8 ± 0.07	11 ± 0.16	10 ± 0.12	13 ± 0.18	12 ± 0.16	13 ± 0.21	12 ± 0.48	14 ± 0.16	12 ± 0.35
Main stem height	15 ± 0.54	16 ± 0.95	16 ± 0.66	20 ± 0.61	16 ± 0.19	19 ± 0.35	18 ± 0.11	20 ± 0.35	19 ± 0.43
Number of fruits per plant	35± 0.38	36± 0.44	50± 0.21b	28± 0.39	24± 0.11	41± 0.25b	38± 0.16	26± 0.21	50± 0.32b
Single fruits weight	14.20± 0.12	15.80± 0.31	19.50±0.47a	28.90±0.43	27.80±0.24	37.50±1.08a	25.7±0.51	22.5±1.04	39.8±1.15a
Plant height	68 ± 0.26	70 ± 1.14	68 ± 1.31	62 ± 1.38	60 ± 1.54	65 ± 1.67	65 ± 1.58	60 ± 2.05	62 ± 1.93
Plant size	60 ± 0.45	61 ± 0.21	62 ± 0.34	51 ± 1.21	52 ± 0.98	84 ± 2.13b	62 ± 1.81	57 ± 0.97	75 ± 2.10b
Fruit length	16.80±0.87	18.00±.64	21.20 ±a1.03	20.50±1.25	22.50±0.83	23.80 ±a1.02	17.20±.36	15.80±0.77	20.00 ±a1.04
Fruit width	1.40±0.02	1.60±0.05	1.80±0.03	2.70±0.06	2.60±0.03	2.85±0.04	2.40±0.05	2.20±0.02	3.00±0.04
Pulp thickness	0.18±0.012	0.24±0.022	0.20±0.019	0.28±0.016	0.25±0.013	0.26±0.007	0.25±0.015	0.26±0.008	0.30±0.011

a Significant value (*P* ≤ 0.05)

b Hightly significant value (*P* ≤ 0.01)

### The mRNA-seq Analysis

The raw reads range from 45,318,018 to 72,847,636 was obtained from all the sequenced samples. The clean data of each sample reached 6.10 Gb averagely. After quality control, the clean reads ranging from 22,659,009 to 36,423,818 was achieved for all samples (Table S1). Furthermore, the numbers of total reads obtained were ranging from 40,887,338 to 72,478,354 (Table S2). The numbers of mapped reads ranged from 39,947,495 to 66,691,290 and the mapped ratios ranged from 38,446,923 (92.98%) to 66,768,777 (91.66%). The relative efficiency between reads and the reference genome was between 85.29% to 93.23%. The unique mapped reads of the samples ranged from 35,495,567 (86.81%) to 46,466,622 (88.95%). The numbers of reads with multiple locations in the reference genome were from 1,281,424 (3.13%) to 2,316,909 (3.18%).

**Figure 2.**
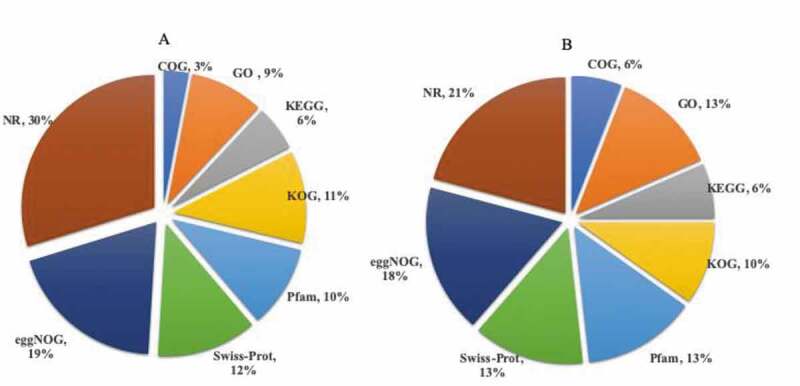
The summary of genes annotated in COG, GO, KEGG, KOG, Pfam, Swiss-Prot, eggNOG, and NR. A: annotated new genes; B: annotated all genes

A total of 36,172 genes were identified and annotated in the public databases (Fig. 2a). Besides new gene annotation, all genes annotation had been carried out. The 212,795 genes were assigned into 8 databases which are COG (12,623), GO (26,716), KEGG (13,848), KOG (20,973), Pfam (28,508), Swiss-Prot (27,994), eggNOG (37,677), and NR (44,456) (Fig. 2b). In all samples, a total of 9117 genes were annotated with the GO database (Table S3). In the flower bud, young fruit stage-1, and young fruit stage-2 tissues, the number of DEGs annotated in the GO database was 3984, 2136, and 2997, respectively. Besides that, the classification and statistics of GO annotations for DEGs were calculated and plotted (Fig. S3). For KEGG annotation, a total of 1848, 1147, and 1442 genes from flower bud, young fruit stage-1, and young fruit stage-2 tissue had been annotated, respectively (Table S4).

### The mRNA Differential Expression Genes (Degs) Analysis

**Figure 3.**
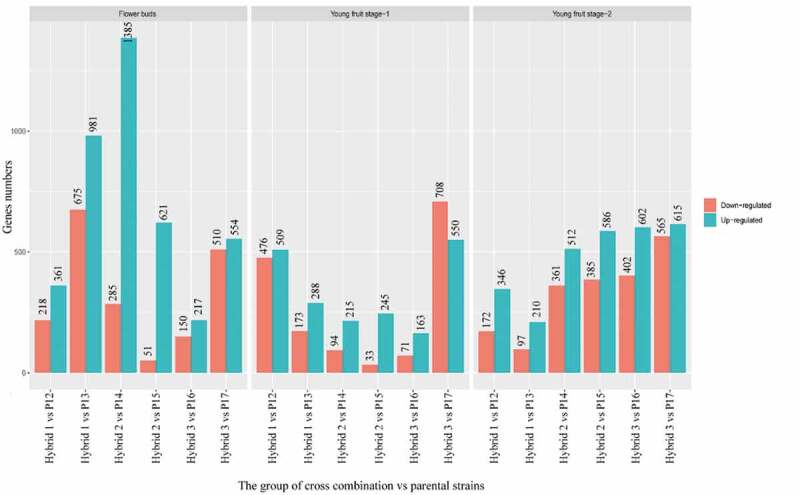
The summary of DEGs in flower bud, young fruit stage-1 and young fruit stage-2 versus three hybrid groups

From the analysis, flower buds, young fruit stage-1, and young fruit stage-2 have a total of 6,008, 3,525, and 4,853 DEGs respectively. The number of up-regulated and down-regulated DEGs in the flower bud category in the three hybrid groups was 4,119 and 1,889. The young fruit stage-1 have 1,970 up-regulated and 1,555 down-regulated DEGs while the young fruit stage-2 category posted 2,871 up-regulated and 1,982 down-regulated DEGs (Fig. 3). From the analysis, Hybrid 2 vs P14 has a higher number of up-regulated DEGs (1385 genes). Overall DEGs showed up-regulation in all hybrid combinations except for the group of Hybrid 3 vs P17 in young fruit stage- Through volcano plot (Fig. S1), the difference of gene expression level and statistical significance between two groups of samples can be observed. A volcano plot was plotted for the three hybrid groups versus parental plants in a flower bud, young fruit stage-1, and young fruit stage-2.

The analysis of the over-presentation of differentially expressed genes in a pathway is the pathway enrichment analysis of DEGs. The hypergeometric test was used to identify the pathway which was significantly enriched in DEGs compared with the whole genome background. In the flower bud, young fruit stage-1 and young fruit stage-2 tissue, a total of 716 up-regulated and 311 down-regulated, 320 up-regulated and 294 down-regulated, 475 up-regulated and 312 down-regulated genes were annotated respectively (Table 3). The KEGG pathway enrichment scatters map of the hybrid groups versus its parental plant in fruit bud, young fruit stage-1 and young fruit-stage 2 was plotted (Fig. S2) to analyze the ratio of the proportion of genes annotated to a pathway in a DEGs to the proportion of genes annotated to that pathway in all genes. The higher the enrichment factor, the more significant the enrichment level of DEGs in those pathways.

**Table 3.** The summary of enriched KEGG pathway in the DEGs

Categories	Hybrid groups vs Parental strains	Up-regulated	Down-regulated
Flower bud	Hybrid 1 vs P12	29	36
	Hybrid 1 vs P13	173	157
	Hybrid 2 vs P14	206	29
	Hybrid 2 vs P15	75	6
	Hybrid 3 vs P16	204	58
	Hybrid 3 vs P17	29	25
Young fruit stage 1	Hybrid 1 vs P12	73	107
	Hybrid 1 vs P13	48	25
	Hybrid 2 vs P14	25	33
	Hybrid 2 vs P15	25	2
	Hybrid 3 vs P16	31	15
	Hybrid 3 vs P17	118	112
Young fruit stage 2	Hybrid 1 vs P12	48	22
	Hybrid 1 vs P13	28	12
	Hybrid 2 vs P14	86	71
	Hybrid 2 vs P15	87	65
	Hybrid 3 vs P16	118	53
	Hybrid 3 vs P17	108	89

### Candidate KEGG Pathways Involving in Regulation of Heterosis

11 KEGG pathways were annotated with higher genes regulated number (Table 4). These KEGG pathways were steroid biosynthesis signaling pathway (Ko00100), photosynthesis signaling pathway (Ko00195), carbon fixation in photosynthetic organism pathway (ko00710), porphyrin and chlorophyll metabolism signaling pathway (ko00860), terpenoid backbone biosynthesis pathway (ko00900), brassinosteroid biosynthesis pathway (ko00905), phenylpropanoid biosynthesis pathway (ko00940), flavonoid biosynthesis pathway (ko00941), plant-hormone signal transduction pathway (ko04075), plant-pathogen interaction (ko04626), and circadian rhythm-plant (ko04712). The pathways mention above play a key role in the biological maintenance of the plant.

**Table 4.** The list of 11 KEGG pathway involving in heterosis

	KEGG ID	Pathway descriptions	Genes number
1	ko00100	Steroid biosynthesis	36
2	ko00195	Photosynthesis	38
3	ko00710	Carbon fixation in photosynthetic	29
4	ko00860	Porphyrin and chlorophyll metabolism	43
5	ko00900	Terpenoid backbone biosynthesis	32
6	ko00905	Brassinosteroid biosynthesis	23
7	ko00940	Phenylpropanoid biosynthesis	48
8	ko00941	Flavonoid biosynthesis	45
9	ko04075	Plant-hormone signal transduction	40
10	ko04626	Plant-pathogen interaction	32
11	ko04712	Circadian rhythm-plant	27

### The miRNA-seq Analysis

A total of 462.54 Mb clean reads were obtained from 27 samples and the numbers of raw reads and clean reads generated from all the samples were from 13,639,625 to 26,723,602 and 12,132,221 to 25,632,680 respectively (Table 5).

**Table 5.** Summary of miRNA-sequencing assembly

Samples	Raw reads	Length<18	Length>30	Clean reads	Q30 (%)	GC (%)
S01	19,497,896	2,216,989	824,152	16,456,755	98.84	51.6
S02	17,853,525	363,744	1,921,758	15,568,023	98.93	50.74
S03	16,875,832	1,726,792	734,673	14,414,367	98.94	51.44
S04	16,031,964	154,534	919,234	14,958,196	98.98	46.47
S05	16,948,197	1,179,950	223,036	15,545,211	99.07	47.77
S06	16,565,027	460,927	385,578	15,718,522	99.02	46.85
S07	21,208,300	267,541	540,027	20,400,732	99.03	47.09
S08	20,622,120	286,874	496,681	19,838,565	99.08	46.94
S09	26,723,602	296,810	794,112	25,632,680	99.05	46.99
S10	19,515,750	179,458	488,247	18,848,045	99.08	47.26
S11	18,388,568	799,586	458,686	17,130,296	99.06	48.31
S12	20,526,049	326,221	359,326	19,840,502	99.06	47.67
S13	23,040,732	1,301,528	911,453	20,827,751	99.05	48.68
S14	23,725,252	406,339	816,963	22,501,950	98.93	47.82
S15	16,087,377	245,929	507,490	15,333,958	99.05	48.08
S16	13,737,257	169,525	526,999	13,040,733	99.13	47.27
S17	16,127,083	215,043	450,577	15,461,463	99.08	47.31
S18	16,122,482	500,817	473,848	15,147,817	99.03	48.05
S19	20,282,292	340,722	929,038	19,012,532	98.87	48.75
S20	18,558,867	426,799	683,126	17,448,942	99.14	48.41
S21	19,433,995	221,467	721,849	18,490,679	98.85	47.99
S22	18,971,363	647,191	606,490	17,717,682	99.07	48.49
S23	18,732,050	712,981	352,837	17,666,232	98.85	48.72
S24	18,844,638	400,344	879,799	17,563,922	99.26	48.3
S25	14,312,429	1,006,835	1,172,965	12,132,221	99.12	48.45
S26	13,639,625	191,078	616,129	12,832,418	99.37	47.34
S27	14,204,248	172,749	1,023,888	13,007,611	99.32	48.14

The *Capsicum annuum* genome (Zunla-1_v2.0) was used as a reference genome for sequence alignment and subsequent analysis. Unannotated reads are aligned with the reference genome using Bowtie2 software to obtain the location information on the reference genome. The number of total reads was ranging from 10,290,313 to 23,951,429, and the mapped reads were 6,071,143 (59%) to 16,488,040 (68.84%).

### Identification of Conserved and Novel miRNA

**Figure 4.**
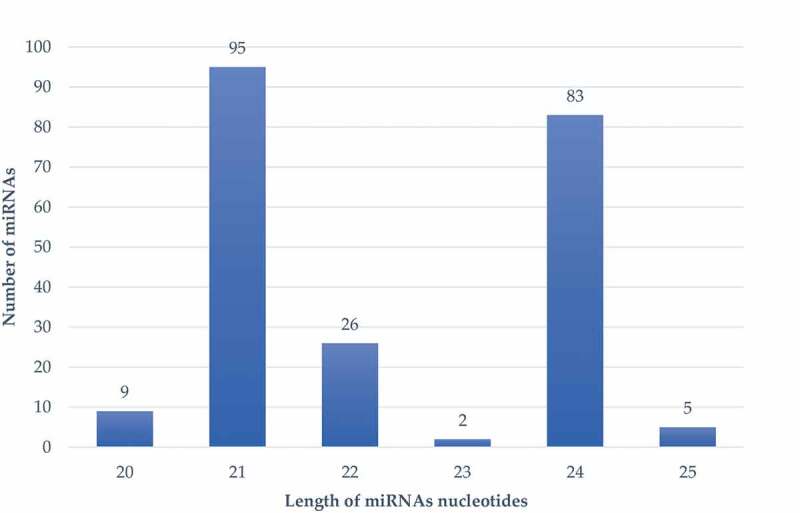
The summary of the miRNA nucleotides length distribution versus the number of miRNAs

A total of 220 miRNAs were identified in all samples, and both 220 miRNAs were novel miRNAs. The number of miRNAs with the length of 21 and 24 nucleotides was 95 and 83, respectively in all the test samples (Fig. 4). The 21 and 24 nucleotides have higher miRNAs number. MicroRNAs are highly conservative among species, based on the sequence similarity, there are 51 miRNAs had been detected in all the samples.

### Differential Expression (Degs) Analysis of miRNA

**Figure 5.**
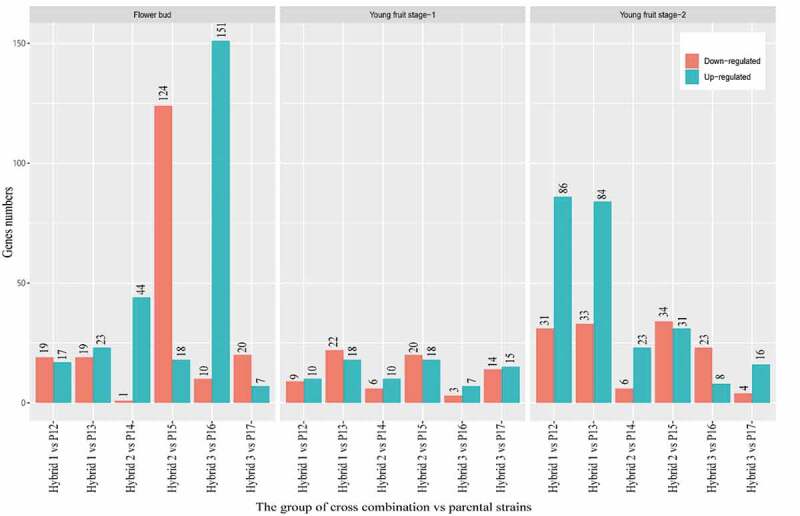
The summary of differential expressed miRNAs of flower bud, young fruit stage-1 and young fruit stage-2 in three hybrid groups vs parental plant

The number of differentials expressed miRNAs of flower bud, young fruit stage-1, and young fruit stage-2 for three hybrid groups (Hybrid 1, Hybrid 2, and Hybrid 3) was 453, 152, and 379, respectively (Fig. 5). The number of up-regulated and down-regulated miRNAs in the flower bud group in three hybrid groups was 260 and 193. In the young fruit stage-1 category for three hybrid groups, a total of 78 up-regulated and 74 down-regulated miRNAs. A total of 248 up-regulated and 131 down-regulated differentials expressed miRNAs were detected in the young fruit stage-2 category. Volcano plots were plotted for the three hybrid groups versus parental plants in a flower bud, young fruit stage-1, and young fruit stage-2 to examine the differences in the expression levels of miRNA and the statistical significance of the differences (Fig. S4).

### The miRNA Target Gene Prediction and Enrichment Analysis

A total of 220 novel miRNA were predicted; of these, 183 predicted miRNAs were successfully predicted with 1440 target genes. In the 1440 target genes, a total of 1427 genes were annotated. Furthermore, these target genes were carried out for GO analysis for functional classification. The defense response (GO:0006952, 96 genes), regulation of transcription DNA-templated (GO:0006355, 93 genes), and response to salt stress (GO:0009651, 94 genes) were the top 3 classifications in a biological process. The nucleus (GO:0005634, 263 genes), plasma membrane (GO:0005886, 226 genes), and plasmodesma (GO:0009506, 150 genes) were the top 3 in cellular component classification. The top 3 molecular function classification were ATP binding (GO:0005524, 127 genes), the binding (GO:0005488), and protein binding (GO:0005515, 222 genes). In flower buds, the 137 pathways were annotated for three hybrid groups versus parental plants (Table 6). A total of 51 and 131 pathways were annotated for young fruit stage-1 and young fruit stage-2 category, respectively. The top 5 candidate KEGG pathways that involved in miRNAs regulation was the plant-pathogen interaction pathway (ko04626, 16 genes), ribosome signaling pathway (ko03010, 13 genes), spliceosome pathway (ko03040, 13 genes), the ubiquitin-mediated proteolysis pathway (ko04120, 10 genes), and plant hormone signal transduction pathways (ko04075, 10 genes).

**Table 6.** The summary of the pathway numbers annotated in a flower bud, young fruit stage 1, and young fruit stage 2 of three hybrid groups versus parental strains

Category	Hybrid groups vs parental strains	Number of pathways
Flower bud	Hybrid 1 vs P12	9
	Hybrid 1 vs P13	36
	Hybrid 2 vs P14	4
	Hybrid 2 vs P15	39
	Hybrid 3 vs P16	40
	Hybrid 3 vs P17	9
Young fruit stage 1	Hybrid 1 vs P12	2
	Hybrid 1 vs P13	16
	Hybrid 2 vs P14	2
	Hybrid 2 vs P15	17
	Hybrid 3 vs P16	1
	Hybrid 3 vs P17	13
Young fruit stage 2	Hybrid 1 vs P12	40
	Hybrid 1 vs P13	36
	Hybrid 2 vs P14	11
	Hybrid 2 vs P15	25
	Hybrid 3 vs P16	11
	Hybrid 3 vs P17	8

### The mRNA-miRNA Integrated Analysis

The differentially expressed miRNAs and genes in two groups were searched, and the relationship between differentially expressed miRNAs and DEGs was searched according to the regulation effect of miRNAs on the mRNA. According to the negative regulatory effect of miRNAs on the mRNA, the miRNAs and mRNA pairs with negative regulatory network relationships were mainly analyzed. From the analyzed data, 84 novel miRNAs were founded to be paired and negatively regulated the genes (Table 7). We found that the scarecrow-like protein 6 and Della protein were regulated by few miRNAs. The scarecrow-like 6 protein was regulated by mir8, mir120, mir184, mir214, mir125, and mir130. The function of Della protein was regulated by mir24, mir74, mir94, mir139, and mir190. The regulation of scarecrow-like protein improved and enhanced the root development while the Della protein maintains and keeping the height of the hot pepper plant. Besides that, the Della protein also functions in stem growth and induced gemination.

**Table 7.** The summary of miRNA-mRNA integrated analysis

miRNA ID	Gene ID	Description
novel_miR8	Capana01g000561	scarecrow-like protein 6
novel_miR11	Capana03g000066	pumilio homolog 23-like isoform X2
novel_miR13	Capana08g001970	F-box/FBD/LRR-repeat protein At1g13570-like
novel_miR14	Capana04g000349	5-nucleotidase domain-containing protein 4 isoforms X1
novel_miR16	Capana06g002186	ethylene receptor 2-like isoform X2
novel_miR20	Capana11g002239	putative late blight resistance protein homolog R1B-8 isoform X2
novel_miR21	Capana06g002232	syntaxin-related protein KNOLLE
novel_miR22	Capana07g000709	RRP12-like protein, partial
novel_miR23	Capana03g004271	geraniol 8-hydroxylase-like isoform X2
novel_miR24	Capana00g003286	DELLA protein RGL1-like
novel_miR25	Capana09g001851	pentatricopeptide repeat-containing protein At2g37310
novel_miR27	Capana12g000764	PXMP2/4 family protein 4
novel_miR30	Capana01g002391	phragmoplast orienting kinesin-1 isoform X2
novel_miR36	Capana03g001123	serine/threonine-protein kinase ATM
novel_miR37	Capana09g000122	nucleoid-associated protein At4g30620, chloroplastic-like
novel_miR38	Capana01g002647	squamosa promoter-binding-like protein 12
novel_miR40	Capana08g000014	probable leucine-rich repeat receptor-like protein kinase At1g35710
novel_miR45	Capana01g002647	squamosa promoter-binding-like protein 12
novel_miR46	Capana01g000620	zinc finger protein ZAT2-like
novel_miR48	Capana12g002879	pentatricopeptide repeat-containing protein At4g16390, chloroplastic
novel_miR49	Capana01g002899	vacuolar protein sorting-associated protein 51 homolog
novel_miR54	Capana01g001128	long-chain-alcohol oxidase FAO4A-like
novel_miR55	Capana08g001970	F-box/FBD/LRR-repeat protein At1g13570-like
novel_miR57	Capana12g000360	TMV resistance protein N-like
novel_miR59	Capana07g001586	Serine/threonine-protein kinase
novel_miR65	Capana01g003194	probable protein phosphatase 2 C 40 isoform X2
novel_miR66	Capana03g002381	protein DETOXIFICATION 49
novel_miR68	Capana12g001214	cellulose synthase A catalytic subunit 2 [UDP-forming]-like
novel_miR70	Capana12g001180	probable LRR receptor-like serine/threonine-protein kinase At1g63430
novel_miR72	Capana11g000521	F-box/WD-40 repeat-containing protein At5g21040-like
novel_miR73	Capana01g000586	sulfofructose kinase-like isoform X1
novel_miR74	Capana00g003286	DELLA protein RGL1-like
novel_miR75	Capana08g001894	SWI/SNF complex subunit SWI3B
novel_miR76	Capana03g001147	protein TORNADO 1
novel_miR78	Capana01g000818	Tsw
novel_miR82	Capana03g002381	protein DETOXIFICATION 49
novel_miR87	Capana11g000435	phragmoplast orienting kinesin 2 isoforms X2
novel_miR93	Capana01g003459	probable helicase CHR10
novel_miR94	Capana00g003286	DELLA protein RGL1-like
novel_miR96	Capana11g002190	putative phospholipid-transporting ATPase 9
novel_miR102	Capana01g001129	long-chain-alcohol oxidase FAO4A-like
novel_miR106	Capana08g001970	F-box/FBD/LRR-repeat protein At1g13570-like
novel_miR113	Capana01g002647	squamosa promoter-binding-like protein 12
novel_miR114	Capana01g000452	protein TPX2 isoform X4
novel_miR115	Capana03g002381	protein DETOXIFICATION 49
novel_miR119	Capana12g001214	cellulose synthase A catalytic subunit 2 [UDP-forming]-like
novel_miR120	Capana01g000561	scarecrow-like protein 6
novel_miR126	Capana11g002190	putative phospholipid-transporting ATPase 9
novel_miR128	Capana00g003683	5-nucleotidase domain-containing protein DDB_G0275467 isoform X1
novel_miR131	Capana01g002647	squamosa promoter-binding-like protein 12
novel_miR134	Capana11g001779	carotenoid 9,10-cleavage dioxygenase-like isoform X2
novel_miR135	Capana03g001123	serine/threonine-protein kinase ATM
novel_miR138	Capana00g003286	DELLA protein RGL1-like
novel_miR147	Capana08g001970	F-box/FBD/LRR-repeat protein At1g13570-like
novel_miR148	Capana11g000534	vicilin-like seed storage protein At2g28490
novel_miR149	Capana02g001786	zingipain-2-like
novel_miR151	Capana12g001214	cellulose synthase A catalytic subunit 2 [UDP-forming]-like
novel_miR152	Capana01g002557	protein LIM1
novel_miR155	Capana03g002557	putative glycerol-3-phosphate transporter 1
novel_miR160	Capana00g001342	kinesin-4-like
novel_miR163	Capana01g002391	phragmoplast orienting kinesin-1 isoform X2
novel_miR169	Capana03g003544	protein PHLOEM PROTEIN 2-LIKE A10
novel_miR173	Capana11g000521	F-box/WD-40 repeat-containing protein At5g21040-like
novel_miR179	Capana08g001970	F-box/FBD/LRR-repeat protein At1g13570-like
novel_miR180	Capana08g001954	separase
novel_miR184	Capana01g000561	scarecrow-like protein 6
novel_miR185	Capana06g001131	pentatricopeptide repeat-containing protein At1g77405
novel_miR186	Capana11g000040	P-loop NTPase domain-containing protein LPA1 homolog 2-like
novel_miR190	Capana00g003286	DELLA protein RGL1-like
novel_miR194	Capana03g000988	RNA-dependent RNA polymerase 2
novel_miR195	Capana03g002215	short-chain dehydrogenase TIC 32, chloroplastic-like
novel_miR196	Capana04g002625	origin of replication complex subunit 1A-like
novel_miR198	Capana06g002170	protein INVOLVED IN DE NOVO 2
novel_miR203	Capana12g001214	uncharacterized protein LOC107875796
novel_miR205	Capana08g001970	F-box/FBD/LRR-repeat protein At1g13570-like
novel_miR213	Capana01g003194	probable protein phosphatase 2 C 40 isoform X2
novel_miR214	Capana01g000561	scarecrow-like protein 6
novel_miR215	Capana01g000561	scarecrow-like protein 6
novel_miR216	capana03g003544	protein PHLOEM PROTEIN 2-LIKE A10
novel_miR220	Capana12g000132	photosystem II 5 kDa protein, chloroplastic-like
novel_miR222	Capana09g000876	transcription factor TCP4-like
novel_miR226	Capana01g001128	long-chain-alcohol oxidase FAO4A-like
novel_miR230	Capana01g000561	scarecrow-like protein 6
novel_miR234	Capana01g002647	squamosa promoter-binding-like protein 12

### The Quantification Analysis of mRNA and miRNA

**Figure 6.**
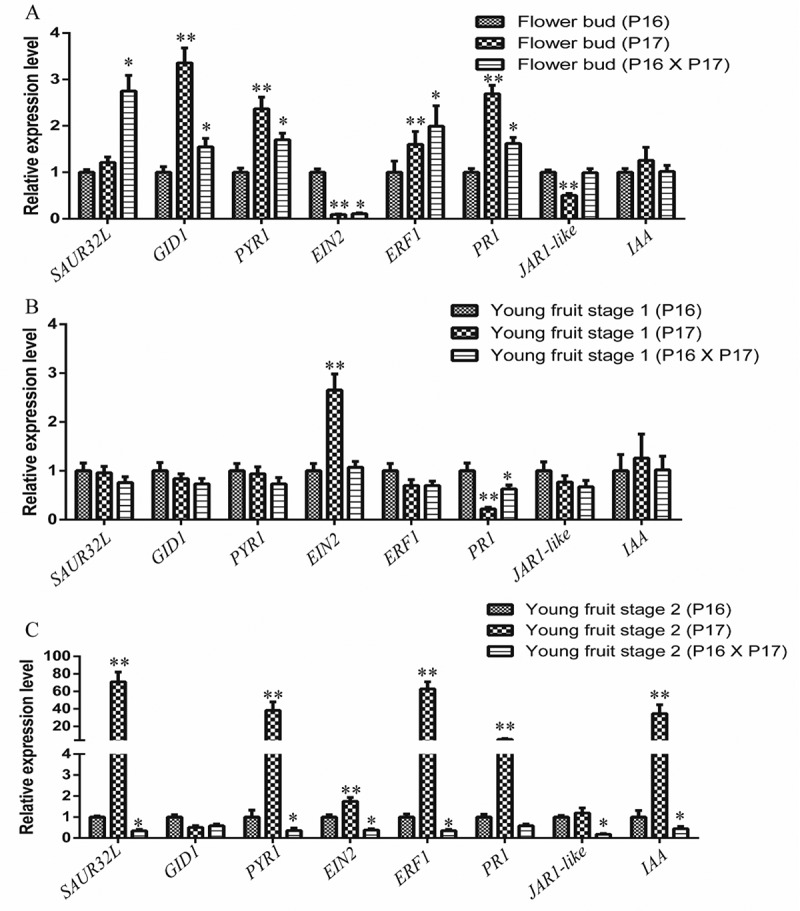
The transcription patterns of *SAUR32L, GID1, PYR1, EIN2. ERF1, PRP1, JAR1-like, IAA* in (a) flower buds, (b) young fruit stage 1, and (c) young fruit stage 2. The significantly differences of hybrid P16 x P17 compare to parent P16 are marked with *, while the significant differences of parent P17 compare to parent P16 are marked with **

The transcription of *SAUR32L, GID1, PYR1, EIN2. ERF1, PR1, JAR1-like, IAA* in the flower bud, young fruit stage 1, and young fruit stage 2 were analyzed in the parent plant P16, parent plant P17, and hybrid cross of P16 x P17 (Fig. 6). The transcription levels in the parent plant P16 was used as the standard against which the relative transcription values at the other stages of development were calculated. In the flower bud of parent P17, the *GID1, PYR1, EIN2, ERF1, PR1*, and *JAR1-like* were significant differences compare to parent P16 and the value of transcription level was 3.36, 2.37, 0.09, 1.60, 2.69, 0.501 respectively. The transcription level of *SAUR32L, GID1, PYR1, EIN2. ERF1* and *PRP1* in the hybrid progeny (P16 x P17) were significant differences compare to parent P16 and the value was 2.75, 1.55, 1.70, 0.11, 1.99, 1.62 respectively. In the young fruit stage 1 of the parent P17, only *EIN2* and *PRP1* were significant differences compare to parent P16 and the value of transcription level was 2.65 and 0.22 respectively. The transcription level of *PRP1* in the hybrid progeny (P16 x P17) in the young fruit stage 1 was significant differences compare to parent P16 and the value was 0.63. For the young fruit stage 2 of the parent P17, the *SAUR32L, PYR1, EIN2. ERF1, PRP1*, and *IAA* were significant differences compare to parent P16 and the level was 70.77, 38.23, 1.75, 62.68, 5.21, and 34.54 respectively. The transcription level of *SAUR32L, PYR1, EIN2. ERF1, JAR1-like*, and *IAA* in the hybrid progeny (P16 x P17) in the young fruit stage 2 were significant differences compare to parent P16 and the value was 0.34, 0.36, 0.38, 0.35, 0.18, and 0.44 respectively.

**Figure 7.**
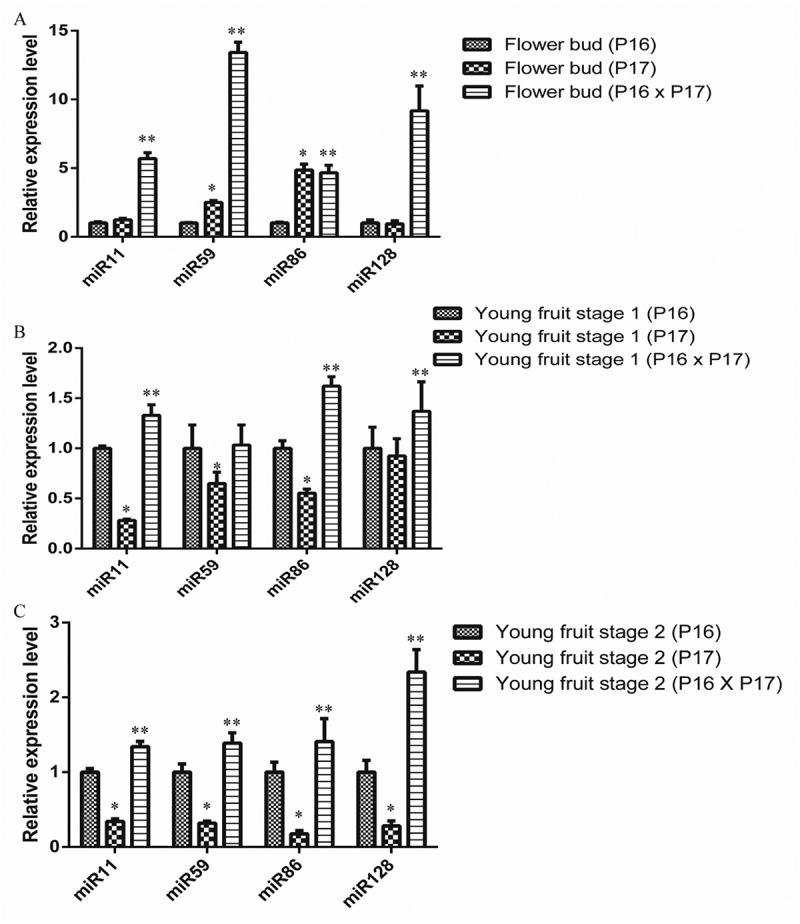
The transcription patterns of miR11, miR59, miR86 and miR128 in (a) flower buds, (b) young fruit stage 1, and (c) young fruit stage 2. The significantly differences of hybrid P16 x P17 compare to parent P16 are marked with **, while the significant differences of parent P17 compare to parent P16 are marked with *

The transcription of miR11, miR59, miR86, and miR128 in the flower bud, young fruit stage 1, and young fruit stage 2 was analyzed in the parent plant P16, parent plant P17, and hybrid cross of P16 x P17 (Fig. 7). The transcription levels in the parent plant P16 was used as the standard against which the relative transcription values at the other stages of development were calculated. The transcription level of miR59 and miR86 in the parent P17 in the flower bud was significant differences compare to parent P16 and the value was 2.50 and 4.85 respectively. The transcription level of miR11, miR59, miR86, and miR128 in the hybrid progeny (P16 x P17) was significant differences compare to parent P16 and the value was 5.69, 13.42, 4.65 and 9.16 respectively. In young fruit stage 1, the expression level of miR11, miR59, miR86 in P17 was 0.28, 0.64, and 0.55 and it showed significant differences compare to parent plant P16; while the expression level of miR11, miR86, and miR128 in the hybrid progeny (P16 x P17) were significant differences compare to parent P16 and the value was 1.32, 1.62, and 1.37 respectively. For the young fruit stage 2, all the miR11, miR59, miR86, and miR128 in parent P17 and hybrid progeny (P16 x P17) showed significant differences compare to parent P16.

## Discussion

In recent years, with the improvement of people’s living standards, consumers are increasingly demanding the quality of peppers, so that researchers pay more attention to the traits of pepper such as early maturity, high yield, and stress resistance.^40–42^ Therefore, it is particularly important to select new pepper varieties with excellent traits.

In this study, the number of fruits per plant, fruit length, and fruit weight was improved in these three hybrids cross combination. However, the mainstem height, plant height, and plant size were not improved. From this hybridization, heterosis only appears in the production of food compare to the physical characteristic of plants. This phenomenon revealed that the hybridization between different parent plants is required to increase and improve the production of hot pepper. The fruit length and fruit weight of the hybrid combination obtained in this study was similar to the findings described by Abu et al, where the heterosis of hot pepper plants can increase fruit length and fruit weight.^43^ A previous study in hot pepper proved that the heterosis could beneficially be enhanced and improved the fruit weight and fruit yield per plant.^44^ The previous researcher revealed that the heterosis to the best parent of 15 intervarietal hybrids of Manzano hot pepper could yield more fruits, improve fruit quality, increase the number of seeds per fruits, seed weight, and locule number.^45^ Likewise, the cross-hybridization between Asian and Ethiopian parents’ plants could significantly improve the number of fruits per plant, dry fruit yield per plant, and the days to maturity. These observations suggested that heterosis breeding could be improving hot pepper to extent of better economic returned.^46^

5 KEGG pathways, phenylpropanoid biosynthesis pathway, flavonoid biosynthesis pathway, and plant-hormone signal transduction pathway were involved in the three groups of hybrid cross combinations. The flavonoid biosynthesis pathway plays a key role in maintaining fertility and protecting the DNA from UV-induced damage. Furthermore, the flavonoids were crucial in maintaining the plant development.^47^ The flavonoid biosynthesis also functions in the fruit ripening process.^48^ A previous study revealed that the flavonoid biosynthesis pathway was involved in the protection of DNAs from UV-induced damage in maize and maintaining fertility in maize and petunia.^49^ Besides that, the phenylpropanoid biosynthesis pathway has up to 48 DEGs among the hybrid cross combination, and it is important in maintaining the success of reproduction. The phenylpropanoid-based polymers such as lignin and suberin are important in maintaining the stability and robustness of gymnosperms and angiosperms from the mechanical and environmental damages.^50^ The plant hormone regulates plant growth, development, and defense across the plant life. In this study, the plant hormone signal transduction pathway was important in heterosis. The ABA and SA are the two major hormone signaling pathway in plants and these pathways might regulate the defense against abiotic and biotic stress. The ABA signaling pathway regulating the plant responses toward environmental stress while the SA signaling pathway modulates the plant immunity to pathogens.^51^ So, the heterosis not only give advantages to fruits production, but it also improved the defense and immunity of hybrid plant so that the hybrid progeny could have higher stress and pathogenic resistant than the parent plant.

The plant hormone signal transduction pathway also holds major DEGs in miRNA. This phenomenon indicates the significance of this pathway in the heterosis of the hot pepper plant. Besides that, the ribosomal signaling pathway was mainly focused on the development of plants. A previous study revealed that some ribosomal proteins were associated with the auxin-related development including cell proliferation, cell expansion, and polarity establishment in leave.^52^ Hence, the miRNA in regulating auxin development through the ribosomal signaling pathway ensures healthy and normal plant development, especially in a hybrid plant. Furthermore, there have several DEGs in the spliceosome signaling pathway where the spliceosomal genes played an important role in plant growth and development. A previous study revealed that the knockdown of splicing protein caused the abnormal stem growth and development in *Arabidopsis*, this suggested that the slicing of an intron is necessary for the normal growth and development of plant.^53^ From the obtained results, the heterosis improved the essential signaling pathway to ensure and maintain the normal growth and development in the hot pepper plant.

In this study, we quantified *SAUR32L, GID1, PYR1, EIN2, ERF1, PR1, JAR1-like*, and *IAA* which we believe it is important in heterosis and maintaining the normal growth of the plant. As a result, these genes showed significant differences by comparing the hybrid progeny toward the parent plant. A previous study revealed that the SAUR genes could directly affect the subfamily II ethylene receptor signaling and induced plant growth and development via the regulation of auxin responses.^54^ Besides that, the SAUR genes are highly transcript in the elongating hypocotyls and the result of overexpression experiments revealed that the SAUR genes could positively regulate the cell expansion to promote hypocotyl growth.^55^ The *IAA was* able to regulate the development and auxin in the plant including cell division, expansion, and differentiation especially in lateral root development.^56^ A previous study revealed that the mutation of *IAA* could inhibit the lateral root primordium development and emergence.^57^ The *GID1* was an endogenous growth regulator that participate in seed germination, seedling growth, flower induction, and development. The mutation of *GID1* could affect reproductive development, the stem length, and fertility of a plant.^58^ The *EIN2* was a central component of the ethylene signaling pathway, the mutation of *EIN2* cause the phenotype in roots, leaves, and flowers.^59^ The activation of *ERF1* could induce defense responses. The previous study revealed that the constitutive transcription of *ERF1* able to improve the resistance of Arabidopsis plants to several species of fungi.^60^ The *JAR1-like* was mainly functioning in the defense against the pathogens and insects. The mutation of *JAR1* can decrease the sensitivity to the jasmonic acid which affected the jasmonate signal transduction.^61^ The *PR1* was transcript as an active plant defense repertoire when the plant was infected with a virulent pathogen. A previous study revealed that the *CaPR-10* in hot pepper could inhibit the oomycete growth and viral infection.^62^

In the mRNA and miRNA integrated study, we found some of the genes which can be regulated by a few miRNAs. Normally, the matching region of miRNAs and mRNA is located in the 2^nd^ to 8^th^ base of the 5ʹ end of miRNAs, which are known as the seed region. As long as the seed region could pair with the mRNA complementary, it can start its function. This is the reason why a miRNA can regulate more than one gene. Among them, the scarecrow-like protein, Della gene were highest regulated by few miRNAs. During the plant development process, the plant hormone gibberellins are important in controlling the cell division and coordination of the direction and extent of cell expansion.^63^ At the same time, the scarecrow-like protein was function as a positive regulator to integrating and maintaining the functionality of gibberellins signaling in the root endodermis.^63^ The Della genes functioning in signal transduction, meristem maintenance, and development. The Della gene is a gibberellin nuclear repressor which is a major component in stem elongation initiating at the apical meristem.^64^ Previous research revealed that the mutation of Della genes resulting in a tall and slender plant and it is important in maintaining the height of plant.^64^ In this study, the integration of miRNA and mRNA was mostly function in the maintaining of root development and height of a plant. The regulation of scarecrow-like protein improved and enhanced the root development while the Della protein maintains and keeping the height of the hot pepper plant. This suggested that a healthier root development and a normal height of plants could enhance the efficiency of water and nutrient transportation in the plant.

## Conclusion

In a conclusion, the hybrid combinations give merit in fruit production which improved the number of fruits per plant, single fruit weight, fruit length, and fruit weight. Based on RNA-seq analysis, we found that the phenylpropanoid biosynthesis pathway, flavonoid biosynthesis pathway, plant-hormone signal transduction pathway, ribosomal signaling pathway, and spliceosome pathway played a key role in regulating and maintaining the molecular mechanisms to ensuring the normal growth and development in hot pepper plant. Besides, the scarecrow-like 6 and Della protein can regulate by few miRNAs. This phenomenon showed that scarecrow-like 6 and Della protein play an important role in plant heterosis. This study provides and contributes a new foundation in the understanding of how heterosis regulates and improves crop production. The heterosis research of the hot pepper shall be continuing and the new hybrid of hot pepper shall be introduced and implemented in the plantation sector, to achieve and meet the international market demand, since the heterosis of hot pepper could improve the crop production.

## Supplementary Material

Supplemental Material
