# Supplementary material for: Integration of mRNA and miRNA profiling reveals the heterosis of three hybrid combinations of Capsicum annuum varieties
Source: GM Crops Food. 2021 Jan 7;12(1):224–41. doi: 10.1080/21645698.2020.1852064 (PMC7808418; doi:10.1080/21645698.2020.1852064)
Supplement: Supplemental Material [file KGMC_A_1852064_SM5177.docx]

**Table S1.** The summary of mRNA sequencing data output statistic.

| **Sample-ID** | **Clean reads** | **Clean bases** | **GC Content** | **Q30 (%)** |
| --- | --- | --- | --- | --- |
| T01 | 25,876,688 | 7,698,472,252 | 43.04% | 93.83% |
| T02 | 32,512,136 | 9,687,625,758 | 43.12% | 93.69% |
| T03 | 29,396,542 | 8,782,203,032 | 43.10% | 93.06% |
| T04 | 27,755,408 | 8,273,908,958 | 43.20% | 93.38% |
| T05 | 31,305,958 | 9,325,233,266 | 42.98% | 93.63% |
| T06 | 28,588,463 | 8,513,632,106 | 42.65% | 93.29% |
| T07 | 30,721,174 | 9,152,884,190 | 43.36% | 93.45% |
| T08 | 28,709,475 | 8,568,851,378 | 42.94% | 93.30% |
| T09 | 28,826,612 | 8,559,627,466 | 43.10% | 93.48% |
| T10 | 36,423,818 | 10,841,592,202 | 42.72% | 93.10% |
| T11 | 32,763,864 | 9,770,292,372 | 42.47% | 92.67% |
| T12 | 29,147,726 | 8,687,510,120 | 42.62% | 93.14% |
| T13 | 33,823,559 | 10,082,949,226 | 43.02% | 93.25% |
| T14 | 30,991,192 | 9,234,526,072 | 43.65% | 92.84% |
| T15 | 29,465,882 | 8,792,084,640 | 43.98% | 93.04% |
| T16 | 29,678,066 | 8,831,535,246 | 43.05% | 93.68% |
| T17 | 23,418,896 | 6,987,537,738 | 42.76% | 93.98% |
| T18 | 23,168,888 | 6,898,216,400 | 42.88% | 94.21% |
| T19 | 35,836,074 | 10,718,605,714 | 43.69% | 93.58% |
| T20 | 28,327,837 | 8,412,161,274 | 43.30% | 94.50% |
| T21 | 31,030,941 | 9,265,267,312 | 43.08% | 93.89% |
| T22 | 36,239,177 | 10,811,099,940 | 42.78% | 93.07% |
| T23 | 36,203,703 | 10,774,144,332 | 42.92% | 93.42% |
| T24 | 34,030,145 | 10,119,591,350 | 42.63% | 93.45% |
| T25 | 29,043,963 | 8,657,432,424 | 42.80% | 93.67% |
| T26 | 32,721,008 | 9,779,734,194 | 43.63% | 93.51% |
| T27 | 33,216,112 | 9,885,920,480 | 42.69% | 93.48% |
| T28 | 29,853,962 | 8,904,879,120 | 44.05% | 93.58% |
| T29 | 28,857,574 | 8,595,376,252 | 44.14% | 93.81% |
| T30 | 32,284,519 | 9,613,318,596 | 43.25% | 93.62% |
| T31 | 26,622,318 | 7,930,661,710 | 43.44% | 94.61% |
| T32 | 24,355,874 | 7,252,284,670 | 43.44% | 94.73% |
| T33 | 25,624,292 | 7,629,371,944 | 43.05% | 94.01% |
| T34 | 30,483,799 | 9,096,522,890 | 44.69% | 94.11% |
| T35 | 27,237,860 | 8,139,734,694 | 43.81% | 93.64% |
| T36 | 24,631,447 | 7,344,871,902 | 43.26% | 93.97% |
| T37 | 26,894,538 | 8,012,599,130 | 43.28% | 94.17% |
| T38 | 30,526,537 | 9,066,400,236 | 43.92% | 93.79% |
| T39 | 25,228,721 | 7,486,266,342 | 44.59% | 94.16% |
| T40 | 24,022,461 | 7,158,286,268 | 44.32% | 94.27% |
| T41 | 26,476,548 | 7,879,252,486 | 43.48% | 94.98% |
| T42 | 31,480,420 | 9,393,265,890 | 43.22% | 94.57% |
| T43 | 30,053,518 | 8,951,769,074 | 43.32% | 94.49% |
| T44 | 26,912,413 | 8,037,251,374 | 43.04% | 94.37% |
| T45 | 27,248,060 | 8,128,532,334 | 43.10% | 94.46% |
| T46 | 26,931,789 | 8,026,960,414 | 43.48% | 94.55% |
| T47 | 27,674,650 | 8,266,020,414 | 43.59% | 94.47% |
| T48 | 26,640,455 | 7,930,297,244 | 43.45% | 94.81% |
| T49 | 28,513,693 | 8,496,643,834 | 43.98% | 94.67% |
| T50 | 31,826,278 | 9,522,279,356 | 44.12% | 93.88% |
| T51 | 29,458,281 | 8,795,140,604 | 43.52% | 93.93% |
| T52 | 24,002,602 | 7,158,579,596 | 43.70% | 94.46% |
| T53 | 27,772,850 | 8,280,101,598 | 43.82% | 94.42% |
| T54 | 27,644,075 | 8,241,502,748 | 43.12% | 94.14% |
| T55 | 25,940,531 | 7,729,081,406 | 43.63% | 94.53% |
| T56 | 25,378,450 | 7,561,106,622 | 43.50% | 94.52% |
| T57 | 24,537,874 | 7,319,023,712 | 43.65% | 94.65% |
| T58 | 27,239,082 | 8,126,014,538 | 43.49% | 94.54% |
| T59 | 24,915,485 | 7,432,496,500 | 43.74% | 94.33% |
| T60 | 24,193,522 | 7,218,749,752 | 43.77% | 94.27% |
| T61 | 24,134,904 | 7,191,353,988 | 43.48% | 94.40% |
| T62 | 25,661,349 | 7,650,815,312 | 43.51% | 93.89% |
| T63 | 26,061,231 | 7,768,074,498 | 44.54% | 94.29% |
| T64 | 25,989,671 | 7,745,081,908 | 43.45% | 94.02% |
| T65 | 24,551,212 | 7,310,687,934 | 43.50% | 94.36% |
| T66 | 26,526,626 | 7,904,828,210 | 43.78% | 94.24% |
| T67 | 22,659,009 | 6,756,741,426 | 44.16% | 94.07% |
| T68 | 20,443,669 | 6,098,899,146 | 43.83% | 94.12% |
| T69 | 25,457,174 | 7,579,798,580 | 44.25% | 94.28% |
| T70 | 22,883,020 | 6,830,473,418 | 43.70% | 94.46% |
| T71 | 24,117,305 | 7,186,813,954 | 43.33% | 94.19% |
| T72 | 20,673,779 | 6,167,239,712 | 43.79% | 94.68% |
| T73 | 25,527,583 | 7,605,805,834 | 44.37% | 94.57% |
| T74 | 30,898,214 | 9,214,025,666 | 43.29% | 94.36% |
| T75 | 30,503,499 | 9,102,100,284 | 43.30% | 93.74% |
| T76 | 27,303,027 | 8,156,450,078 | 44.13% | 94.04% |
| T77 | 29,315,216 | 8,734,460,046 | 44.07% | 94.46% |
| T78 | 29,026,453 | 8,647,968,172 | 43.44% | 94.19% |
| T79 | 25,834,566 | 7,702,827,234 | 45.07% | 94.71% |
| T80 | 27,705,788 | 8,240,104,646 | 43.57% | 94.28% |
| T81 | 24,707,738 | 7,376,817,010 | 44.86% | 94.19% |

*Samples: name of the sample testes; clean reads: total of pair-end reads in clean data; clean bases: total bases number of clean data; GC content: the GC contents in clean data, the percentage of G and C bases in total bases; Q30 (%): the percentage of bases with clean data mass values greater than or equal to 30.

**Table S2.** The summary of mRNA sequence alignment results between sample sequencing data and selected reference genomes.

| Samples-ID | Total Reads | Mapped Reads | Unique Mapped Reads | Multiple Map Reads | Reads Map to '+' | Reads Map to '-' |
| --- | --- | --- | --- | --- | --- | --- |
| T01 | 51,753,376 | 48,122,548 (92.98%) | 46,536,151 (89.92%) | 1,586,397 (3.07%) | 23,846,511 (46.08%) | 23,915,989 (46.21%) |
| T02 | 65,024,272 | 60,351,751 (92.81%) | 58,423,021 (89.85%) | 1,928,730 (2.97%) | 29,909,353 (46.00%) | 30,008,048 (46.15%) |
| T03 | 58,793,084 | 53,729,096 (91.39%) | 51,998,967 (88.44%) | 1,730,129 (2.94%) | 26,621,086 (45.28%) | 26,729,323 (45.46%) |
| T04 | 55,510,816 | 51,079,986 (92.02%) | 49,411,838 (89.01%) | 1,668,148 (3.01%) | 25,309,932 (45.59%) | 25,391,601 (45.74%) |
| T05 | 62,611,916 | 57,548,156 (91.91%) | 55,571,632 (88.76%) | 1,976,524 (3.16%) | 28,508,071 (45.53%) | 28,616,805 (45.71%) |
| T06 | 57,176,926 | 52,652,841 (92.09%) | 50,908,830 (89.04%) | 1,744,011 (3.05%) | 26,074,829 (45.60%) | 26,165,221 (45.76%) |
| T07 | 61,442,348 | 55,268,287 (89.95%) | 53,279,049 (86.71%) | 1,989,238 (3.24%) | 27,312,052 (44.45%) | 27,454,382 (44.68%) |
| T08 | 57,418,950 | 51,775,353 (90.17%) | 49,928,569 (86.95%) | 1,846,784 (3.22%) | 25,616,456 (44.61%) | 25,729,148 (44.81%) |
| T09 | 57,653,224 | 53,152,342 (92.19%) | 51,232,659 (88.86%) | 1,919,683 (3.33%) | 26,296,745 (45.61%) | 26,382,368 (45.76%) |
| T10 | 72,847,636 | 66,768,777 (91.66%) | 64,451,868 (88.47%) | 2,316,909 (3.18%) | 33,048,416 (45.37%) | 33,150,437 (45.51%) |
| T11 | 65,527,728 | 59,851,764 (91.34%) | 57,910,040 (88.37%) | 1,941,724 (2.96%) | 29,654,102 (45.25%) | 29,748,899 (45.40%) |
| T12 | 58,295,452 | 53,624,153 (91.99%) | 51,876,459 (88.99%) | 1,747,694 (3.00%) | 26,562,646 (45.57%) | 26,647,419 (45.71%) |
| T13 | 67,647,118 | 61,457,245 (90.85%) | 59,381,280 (87.78%) | 2,075,965 (3.07%) | 30,434,622 (44.99%) | 30,544,902 (45.15%) |
| T14 | 61,982,384 | 54,793,871 (88.40%) | 52,828,137 (85.23%) | 1,965,734 (3.17%) | 27,037,518 (43.62%) | 27,217,673 (43.91%) |
| T15 | 58,931,764 | 51,709,944 (87.75%) | 49,795,435 (84.50%) | 1,914,509 (3.25%) | 25,467,890 (43.22%) | 25,670,934 (43.56%) |
| T16 | 59,356,132 | 54,214,933 (91.34%) | 52,432,203 (88.33%) | 1,782,730 (3.00%) | 26,869,154 (45.27%) | 26,941,314 (45.39%) |
| T17 | 46,837,792 | 42,730,864 (91.23%) | 41,063,898 (87.67%) | 1,666,966 (3.56%) | 21,076,189 (45.00%) | 21,177,083 (45.21%) |
| T18 | 46,337,776 | 42,339,375 (91.37%) | 40,893,313 (88.25%) | 1,446,062 (3.12%) | 20,898,392 (45.10%) | 21,062,822 (45.45%) |
| T19 | 71,672,148 | 63,826,569 (89.05%) | 61,613,633 (85.97%) | 2,212,936 (3.09%) | 31,594,004 (44.08%) | 31,729,875 (44.27%) |
| T20 | 56,655,674 | 51,866,800 (91.55%) | 50,140,557 (88.50%) | 1,726,243 (3.05%) | 25,665,344 (45.30%) | 25,765,384 (45.48%) |
| T21 | 62,061,882 | 57,129,384 (92.05%) | 55,294,753 (89.10%) | 1,834,631 (2.96%) | 28,338,780 (45.66%) | 28,393,826 (45.75%) |
| T22 | 72,478,354 | 66,691,290 (92.02%) | 64,466,622 (88.95%) | 2,224,668 (3.07%) | 33,029,743 (45.57%) | 33,178,287 (45.78%) |
| T23 | 72,407,406 | 66,180,533 (91.40%) | 63,893,062 (88.24%) | 2,287,471 (3.16%) | 32,732,303 (45.21%) | 32,893,788 (45.43%) |
| T24 | 68,060,290 | 63,257,693 (92.94%) | 61,030,132 (89.67%) | 2,227,561 (3.27%) | 31,308,348 (46.00%) | 31,411,333 (46.15%) |
| T25 | 58,087,926 | 53,462,570 (92.04%) | 51,594,203 (88.82%) | 1,868,367 (3.22%) | 26,426,336 (45.49%) | 26,507,365 (45.63%) |
| T26 | 65,442,016 | 58,886,520 (89.98%) | 56,799,213 (86.79%) | 2,087,307 (3.19%) | 29,095,499 (44.46%) | 29,280,331 (44.74%) |
| T27 | 66,432,224 | 61,094,775 (91.97%) | 58,822,519 (88.55%) | 2,272,256 (3.42%) | 30,124,886 (45.35%) | 30,258,641 (45.55%) |
| T28 | 59,707,924 | 52,669,243 (88.21%) | 50,779,789 (85.05%) | 1,889,454 (3.16%) | 26,084,607 (43.69%) | 26,242,491 (43.95%) |
| T29 | 57,715,148 | 50,166,042 (86.92%) | 48,283,497 (83.66%) | 1,882,545 (3.26%) | 24,817,634 (43.00%) | 24,966,169 (43.26%) |
| T30 | 64,569,038 | 59,452,769 (92.08%) | 57,436,848 (88.95%) | 2,015,921 (3.12%) | 29,513,065 (45.71%) | 29,596,265 (45.84%) |
| T31 | 53,244,636 | 48,551,754 (91.19%) | 46,878,786 (88.04%) | 1,672,968 (3.14%) | 24,044,687 (45.16%) | 24,137,811 (45.33%) |
| T32 | 48,711,748 | 45,092,137 (92.57%) | 43,516,229 (89.33%) | 1,575,908 (3.24%) | 22,374,417 (45.93%) | 22,409,060 (46.00%) |
| T33 | 51,248,584 | 47,376,204 (92.44%) | 45,779,731 (89.33%) | 1,596,473 (3.12%) | 23,486,975 (45.83%) | 23,556,252 (45.96%) |
| T34 | 60,967,598 | 53,068,180 (87.04%) | 50,899,173 (83.49%) | 2,169,007 (3.56%) | 26,188,249 (42.95%) | 26,397,383 (43.30%) |
| T35 | 54,475,720 | 48,950,069 (89.86%) | 47,175,539 (86.60%) | 1,774,530 (3.26%) | 24,226,485 (44.47%) | 24,352,744 (44.70%) |
| T36 | 49,262,894 | 45,417,060 (92.19%) | 43,768,605 (88.85%) | 1,648,455 (3.35%) | 22,498,632 (45.67%) | 22,569,808 (45.82%) |
| T37 | 53,789,076 | 49,599,799 (92.21%) | 47,878,923 (89.01%) | 1,720,876 (3.20%) | 24,591,946 (45.72%) | 24,639,970 (45.81%) |
| T38 | 61,053,074 | 54,834,639 (89.81%) | 52,946,902 (86.72%) | 1,887,737 (3.09%) | 27,154,521 (44.48%) | 27,279,812 (44.68%) |
| T39 | 50,457,442 | 43,195,134 (85.61%) | 41,558,412 (82.36%) | 1,636,722 (3.24%) | 21,331,056 (42.28%) | 21,462,745 (42.54%) |
| T40 | 48,044,922 | 42,204,427 (87.84%) | 40,664,139 (84.64%) | 1,540,288 (3.21%) | 20,870,760 (43.44%) | 20,983,760 (43.68%) |
| T41 | 52,953,096 | 48,722,802 (92.01%) | 46,939,135 (88.64%) | 1,783,667 (3.37%) | 24,165,485 (45.64%) | 24,224,335 (45.75%) |
| T42 | 62,960,840 | 57,670,685 (91.60%) | 55,555,513 (88.24%) | 2,115,172 (3.36%) | 28,581,583 (45.40%) | 28,650,148 (45.50%) |
| T43 | 60,107,036 | 55,314,412 (92.03%) | 53,374,392 (88.80%) | 1,940,020 (3.23%) | 27,423,137 (45.62%) | 27,505,418 (45.76%) |
| T44 | 53,824,826 | 49,263,705 (91.53%) | 47,600,868 (88.44%) | 1,662,837 (3.09%) | 24,417,156 (45.36%) | 24,517,270 (45.55%) |
| T45 | 54,496,120 | 49,942,433 (91.64%) | 48,190,330 (88.43%) | 1,752,103 (3.22%) | 24,750,053 (45.42%) | 24,838,233 (45.58%) |
| T46 | 53,863,578 | 49,193,445 (91.33%) | 47,457,098 (88.11%) | 1,736,347 (3.22%) | 24,336,412 (45.18%) | 24,424,516 (45.35%) |
| T47 | 55,349,300 | 51,147,950 (92.41%) | 49,278,829 (89.03%) | 1,869,121 (3.38%) | 25,319,254 (45.74%) | 25,394,873 (45.88%) |
| T48 | 53,280,910 | 49,675,037 (93.23%) | 47,971,183 (90.03%) | 1,703,854 (3.20%) | 24,621,915 (46.21%) | 24,676,174 (46.31%) |
| T49 | 57,027,386 | 51,542,209 (90.38%) | 49,490,484 (86.78%) | 2,051,725 (3.60%) | 25,474,818 (44.67%) | 25,572,422 (44.84%) |
| T50 | 63,652,556 | 56,044,736 (88.05%) | 53,881,593 (84.65%) | 2,163,143 (3.40%) | 27,649,921 (43.44%) | 27,850,981 (43.75%) |
| T51 | 58,916,562 | 53,911,034 (91.50%) | 51,795,639 (87.91%) | 2,115,395 (3.59%) | 26,656,064 (45.24%) | 26,754,375 (45.41%) |
| T52 | 48,005,204 | 43,703,243 (91.04%) | 42,039,753 (87.57%) | 1,663,490 (3.47%) | 21,633,320 (45.06%) | 21,708,608 (45.22%) |
| T53 | 55,545,700 | 49,497,107 (89.11%) | 47,508,153 (85.53%) | 1,988,954 (3.58%) | 24,485,959 (44.08%) | 24,579,286 (44.25%) |
| T54 | 55,288,150 | 50,968,264 (92.19%) | 49,109,006 (88.82%) | 1,859,258 (3.36%) | 25,265,843 (45.70%) | 25,330,052 (45.81%) |
| T55 | 51,881,062 | 47,320,792 (91.21%) | 45,724,535 (88.13%) | 1,596,257 (3.08%) | 23,441,317 (45.18%) | 23,540,948 (45.37%) |
| T56 | 50,756,900 | 47,322,226 (93.23%) | 45,792,632 (90.22%) | 1,529,594 (3.01%) | 23,442,792 (46.19%) | 23,529,600 (46.36%) |
| T57 | 49,075,748 | 45,338,664 (92.39%) | 43,860,267 (89.37%) | 1,478,397 (3.01%) | 22,495,484 (45.84%) | 22,572,270 (45.99%) |
| T58 | 54,478,164 | 50,546,421 (92.78%) | 48,791,748 (89.56%) | 1,754,673 (3.22%) | 25,065,142 (46.01%) | 25,180,835 (46.22%) |
| T59 | 49,830,970 | 46,062,635 (92.44%) | 44,335,454 (88.97%) | 1,727,181 (3.47%) | 22,846,241 (45.85%) | 22,928,492 (46.01%) |
| T60 | 48,387,044 | 44,474,552 (91.91%) | 42,745,866 (88.34%) | 1,728,686 (3.57%) | 21,937,371 (45.34%) | 22,106,023 (45.69%) |
| T61 | 48,269,808 | 44,747,091 (92.70%) | 43,211,986 (89.52%) | 1,535,105 (3.18%) | 22,116,889 (45.82%) | 22,239,230 (46.07%) |
| T62 | 51,322,698 | 47,268,873 (92.10%) | 45,489,714 (88.63%) | 1,779,159 (3.47%) | 23,393,599 (45.58%) | 23,520,194 (45.83%) |
| T63 | 52,122,462 | 46,017,829 (88.29%) | 44,108,488 (84.62%) | 1,909,341 (3.66%) | 22,661,845 (43.48%) | 22,873,669 (43.88%) |
| T64 | 51,979,342 | 47,727,965 (91.82%) | 45,757,220 (88.03%) | 1,970,745 (3.79%) | 23,580,254 (45.36%) | 23,669,844 (45.54%) |
| T65 | 49,102,424 | 45,274,088 (92.20%) | 43,671,413 (88.94%) | 1,602,675 (3.26%) | 22,402,104 (45.62%) | 22,501,527 (45.83%) |
| T66 | 53,053,252 | 48,368,473 (91.17%) | 46,536,017 (87.72%) | 1,832,456 (3.45%) | 23,906,561 (45.06%) | 24,013,160 (45.26%) |
| T67 | 45,318,018 | 39,947,495 (88.15%) | 38,391,009 (84.71%) | 1,556,486 (3.43%) | 19,710,568 (43.49%) | 19,850,262 (43.80%) |
| T68 | 40,887,338 | 36,776,991 (89.95%) | 35,495,567 (86.81%) | 1,281,424 (3.13%) | 18,178,468 (44.46%) | 18,288,780 (44.73%) |
| T69 | 50,914,348 | 44,640,408 (87.68%) | 43,101,057 (84.65%) | 1,539,351 (3.02%) | 22,094,279 (43.39%) | 22,224,299 (43.65%) |
| T70 | 45,766,040 | 42,227,477 (92.27%) | 40,681,237 (88.89%) | 1,546,240 (3.38%) | 20,892,060 (45.65%) | 20,971,883 (45.82%) |
| T71 | 48,234,610 | 44,844,284 (92.97%) | 43,183,640 (89.53%) | 1,660,644 (3.44%) | 22,193,934 (46.01%) | 22,268,789 (46.17%) |
| T72 | 41,347,558 | 38,446,923 (92.98%) | 36,935,859 (89.33%) | 1,511,064 (3.65%) | 19,008,546 (45.97%) | 19,088,522 (46.17%) |
| T73 | 51,055,166 | 45,458,708 (89.04%) | 43,711,511 (85.62%) | 1,747,197 (3.42%) | 22,441,897 (43.96%) | 22,615,841 (44.30%) |
| T74 | 61,796,428 | 54,052,480 (87.47%) | 52,236,177 (84.53%) | 1,816,303 (2.94%) | 26,807,483 (43.38%) | 26,898,728 (43.53%) |
| T75 | 61,006,998 | 55,810,718 (91.48%) | 53,614,677 (87.88%) | 2,196,041 (3.60%) | 27,615,438 (45.27%) | 27,718,267 (45.43%) |
| T76 | 54,606,054 | 46,575,054 (85.29%) | 44,555,879 (81.60%) | 2,019,175 (3.70%) | 22,949,311 (42.03%) | 23,105,808 (42.31%) |
| T77 | 58,630,432 | 52,645,799 (89.79%) | 50,572,252 (86.26%) | 2,073,547 (3.54%) | 25,959,878 (44.28%) | 26,124,774 (44.56%) |
| T78 | 58,052,906 | 53,915,921 (92.87%) | 52,032,073 (89.63%) | 1,883,848 (3.25%) | 26,695,026 (45.98%) | 26,781,875 (46.13%) |
| T79 | 51,669,132 | 44,782,556 (86.67%) | 42,928,740 (83.08%) | 1,853,816 (3.59%) | 22,082,616 (42.74%) | 22,254,226 (43.07%) |
| T80 | 55,411,576 | 51,183,609 (92.37%) | 49,275,821 (88.93%) | 1,907,788 (3.44%) | 25,305,508 (45.67%) | 25,426,462 (45.89%) |
| T81 | 49,415,476 | 42,325,795 (85.65%) | 40,406,480 (81.77%) | 1,919,315 (3.88%) | 20,790,495 (42.07%) | 20,965,355 (42.43%) |

*Sample-ID: sample number; total reads: number of clean reads by single end; mapped reads: comparing the number of reads on the reference genome with the percentage in clean Reads; unique mapped reads: number of reads compared to the unique location of the reference genome and percentage in clean Reads; multiple map reads: number of reads compared to multiple locations of the reference genome and percentage in clean reads; reads map to ‘+’: number of reads and percentage of clean reads comparing reference genome positive chains; reads map to ‘-’: number of reads and percentage of clean reads comparing negative chains of reference genomes.

**Table S3.** The summary of annotated DEGs in GO.

| **Categories** | **Hybrids groups vs parental strains** | **Number of genes** |
| --- | --- | --- |
| Flower bud | Hybrid 1 vs P12 | 315 |
|  | Hybrid 1 vs P13 | 1095 |
|  | Hybrid 2 vs P14 | 1026 |
|  | Hybrid 2 vs P15 | 319 |
|  | Hybrid 3 vs P16 | 1050 |
|  | Hybrid 3 vs P17 | 179 |
|  |  |  |
| Young fruit stage 1 | Hybrid 1 vs P12 | 657 |
|  | Hybrid 1 vs P13 | 256 |
|  | Hybrid 2 vs P14 | 160 |
|  | Hybrid 2 vs P15 | 106 |
|  | Hybrid 3 vs P16 | 146 |
|  | Hybrid 3 vs P17 | 811 |
|  |  |  |
| Young fruit stage 2 | Hybrid 1 vs P12 | 298 |
|  | Hybrid 1 vs P13 | 153 |
|  | Hybrid 2 vs P14 | 547 |
|  | Hybrid 2 vs P15 | 614 |
|  | Hybrid 3 vs P16 | 651 |
|  | Hybrid 3 vs P17 | 734 |

**Table S4.** The summary of annotated DEGs in KEGG.

| **Categories** | **Hybrids groups vs parental strains** | **Number of genes** |
| --- | --- | --- |
| Flower bud | Hybrid 1 vs P12 | 145 |
|  | Hybrid 1 vs P13 | 551 |
|  | Hybrid 2 vs P14 | 435 |
|  | Hybrid 2 vs P15 | 137 |
|  | Hybrid 3 vs P16 | 491 |
|  | Hybrid 3 vs P17 | 89 |
|  |  |  |
| Young fruit stage 1 | Hybrid 1 vs P12 | 361 |
|  | Hybrid 1 vs P13 | 124 |
|  | Hybrid 2 vs P14 | 94 |
|  | Hybrid 2 vs P15 | 48 |
|  | Hybrid 3 vs P16 | 77 |
|  | Hybrid 3 vs P17 | 443 |
|  |  |  |
| Young fruit stage 2 | Hybrid 1 vs P12 | 140 |
|  | Hybrid 1 vs P13 | 75 |
|  | Hybrid 2 vs P14 | 265 |
|  | Hybrid 2 vs P15 | 268 |
|  | Hybrid 3 vs P16 | 329 |
|  | Hybrid 3 vs P17 | 365 |


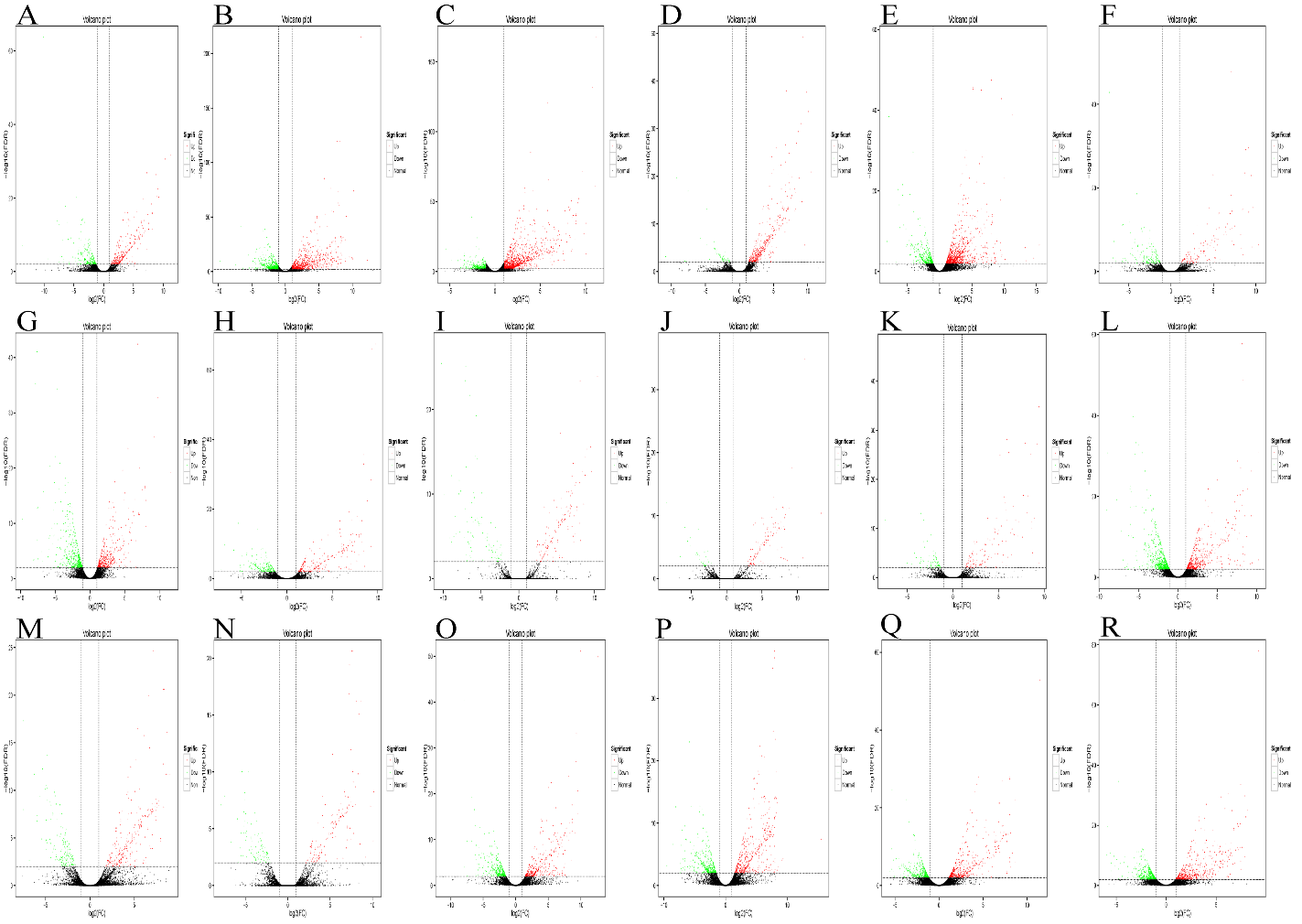


Figure S1. The volcano plot of DEGs for tested samples. Each point in the differential expression volcanic map represents a gene, and the X-axis represents the logarithmic value of the multiple of the DEGs in two samples; the Y-axis represents the negative logarithmic value of the statistical significance of the variation of the gene expression. The greater the X-axis absolute value, the greater the difference of expression multiples between two groups. The larger the ordinate value, the more significant the difference expression, the more reliable the DEGs was screened. The green dots in the volcano plot represent down-regulated DEGS, the red dots represent up-regulated DEGs, and the black dots represent non-DEGs. A-F, G-L, and M-R represents flower bud, young fruit stage-1 and young fruit stage-2 respectively. A, G, M: Hybrid 1 vs P12; B, H, N: Hybrid 1 vs P13; C, I, O: Hybrid 2 vs P14; D, J, P: Hybrid 2 vs P15; E, K, Q: Hybrid 3 vs P16; F, L, R: Hybrid 3 vs P17.


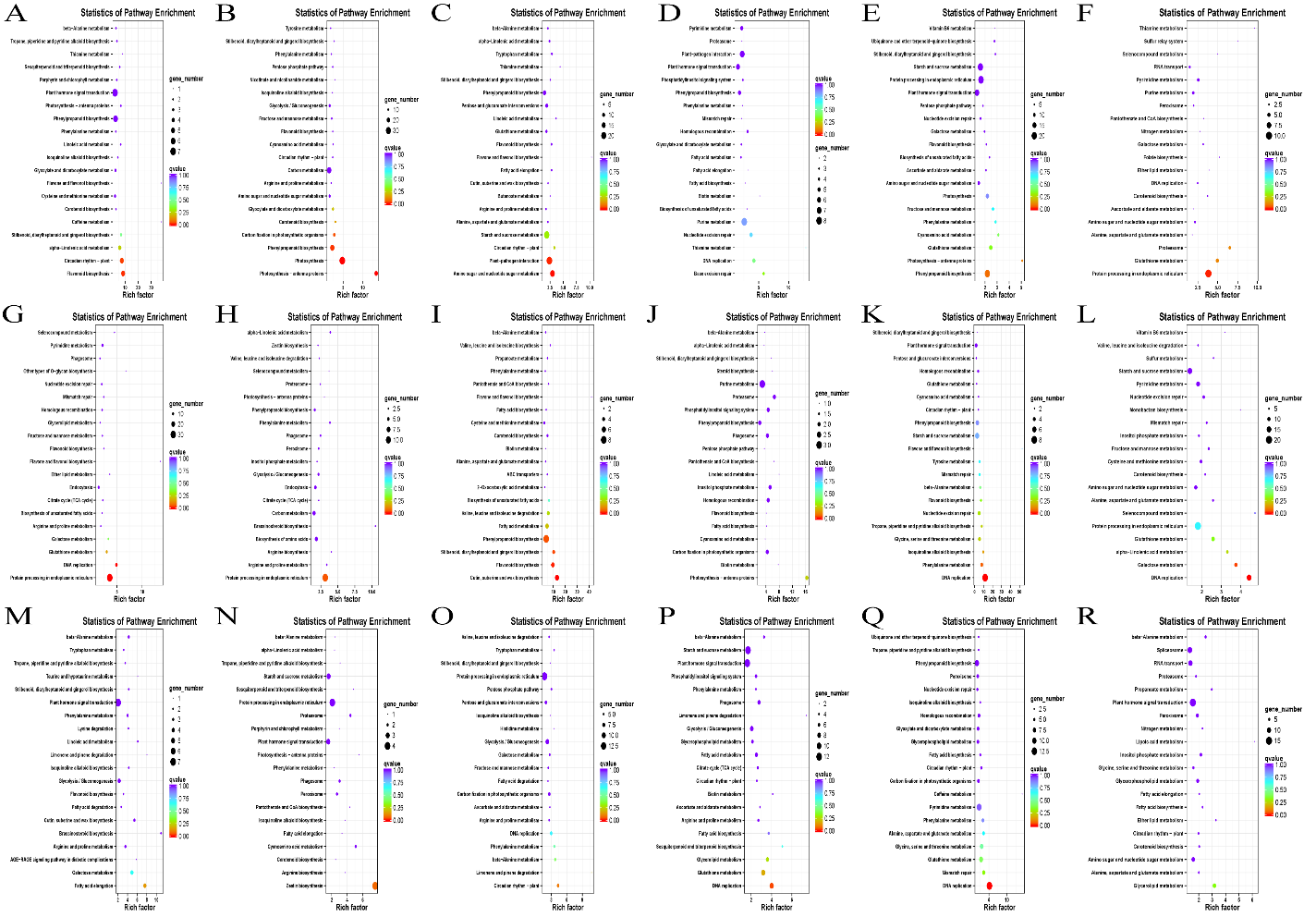


Figure S2. The KEGG pathway enrichment scatter map of tested samples. Each circle in the map represents a KEGG pathway, the Y-axis represents the name of the pathway and the X-axis is the enrichment factor. It represents the ratio of the proportion of genes annotated to a pathway in different genes to the proportion of genes annotated to the pathway in all genes. The higher the enrichment factor is, the more significant the enrichment level of DEGs in this pathway. The smaller the q-value, the more reliable the enrichment significance of DEGs in the pathway. The size of the circle indicates the number of genes enriched in the pathway, and the larger the circle, the more genes there are. A-F, G-L, and M-R represents flower bud, young fruit stage-1 and young fruit stage-2 respectively. A, G, M: Hybrid 1 vs P12; B, H, N: Hybrid 1 vs P13; C, I, O: Hybrid 2 vs P14; D, J, P: Hybrid 2 vs P15; E, K, Q: Hybrid 3 vs P16; F, L, R: Hybrid 3 vs P17.


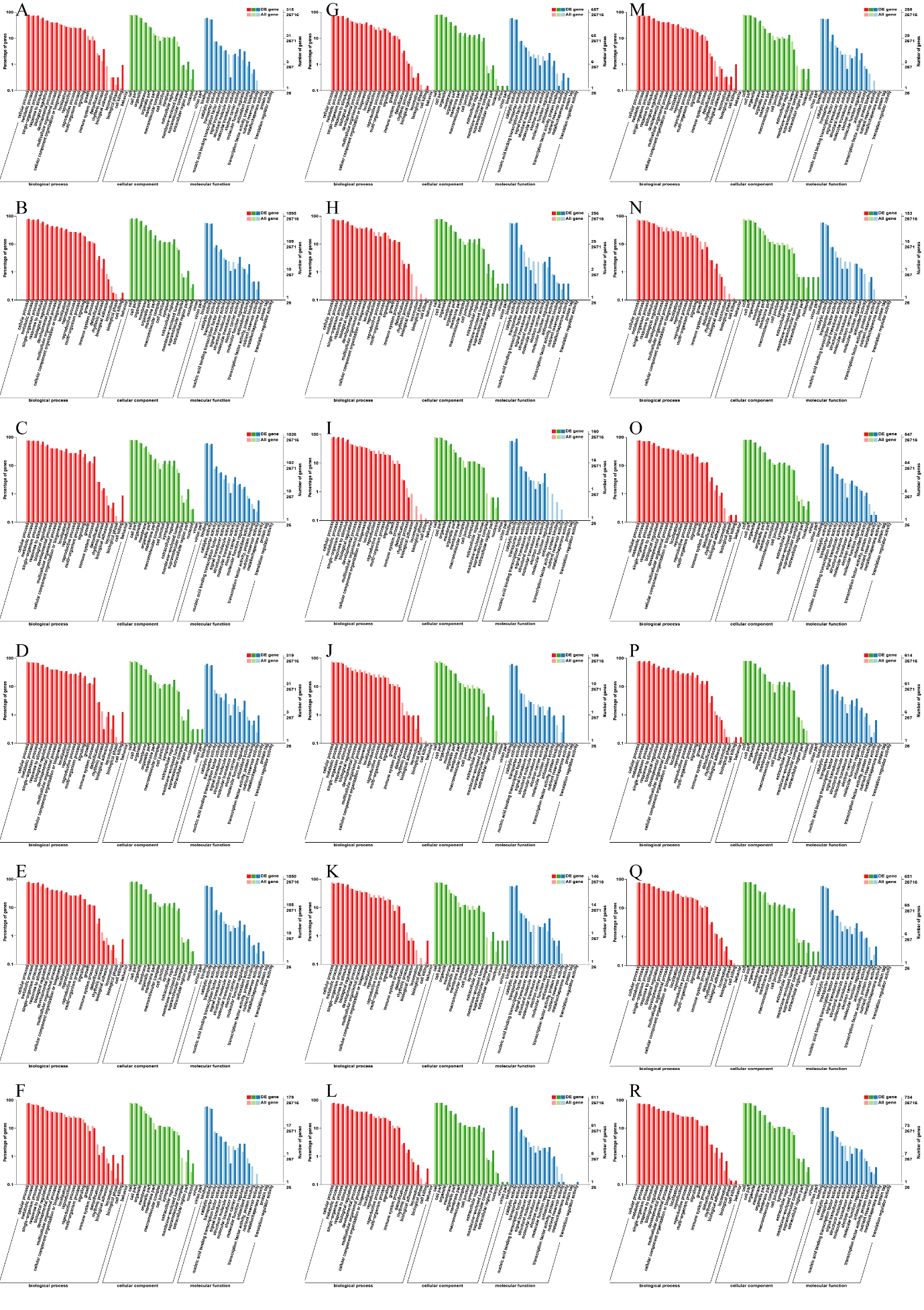


Figure S3. The summary of classification and statistics of GO annotations for DEGs. The X-axis is GO classification, the left and the right of the Y-axis is percentage of the number of genes and the number of genes respectively. The map shows the gene enrichment of the secondary functions of GO in the background of DEGs and in the background of genes. It reflects the status of the secondary functions of GO in the two backgrounds. The secondary functions with obvious proportion differences show that the enrichment trend of DEGs is different from that all genes. A-F, G-L, and M-R represents flower bud, young fruit stage-1 and young fruit stage-2 respectively. A, G, M: Hybrid 1 vs P12; B, H, N: Hybrid 1 vs P13; C, I, O: Hybrid 2 vs P14; D, J, P: Hybrid 2 vs P15; E, K, Q: Hybrid 3 vs P16; F, L, R: Hybrid 3 vs P17.


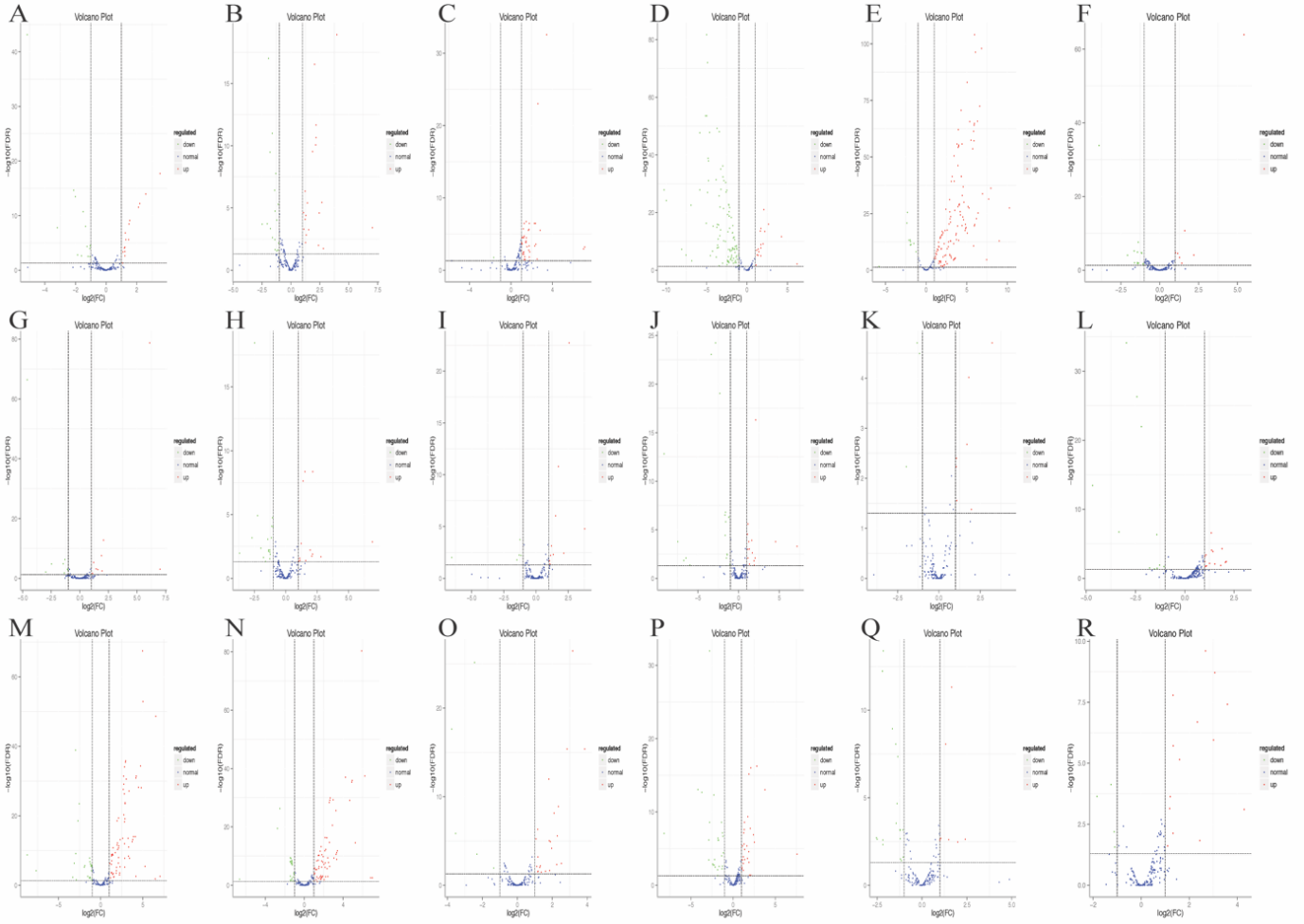


Figure S4. The volcano plot of differential expressed miRNAs for tested samples. Each point in the differential expression volcanic map represents a gene, and the X-axis represents the logarithmic value of the multiple of the DEGs in two samples; the Y-axis represents the negative logarithmic value of the statistical significance of the variation of the gene expression. The greater the X-axis absolute value, the greater the difference of expression multiples between two groups. The larger the ordinate value, the more significant the difference expression, the more reliable the DEGs was screened. The green dots in the volcano plot represent down-regulated miRNAs, the red dots represent up-regulated miRNAs, and the black dots represent non-differentiated miRNA. A-F, G-L, and M-R represents flower bud, young fruit stage-1 and young fruit stage-2 respectively. A, G, M: Hybrid 1 vs P12; B, H, N: Hybrid 1 vs P13; C, I, O: Hybrid 2 vs P14; D, J, P: Hybrid 2 vs P15; E, K, Q: Hybrid 3 vs P16; F, L, R: Hybrid 3 vs P17.
